# An Eye-Movement Analysis of Overt Visual Attention During Consecutive and Simultaneous Interpreting Modes in a Remotely Interpreted Investigative Interview

**DOI:** 10.3389/fpsyg.2022.764460

**Published:** 2022-03-25

**Authors:** Stephen Doherty, Natalie Martschuk, Jane Goodman-Delahunty, Sandra Hale

**Affiliations:** ^1^School of Humanities and Languages, University of New South Wales, Sydney, NSW, Australia; ^2^Griffith Criminology Institute, Griffith University, Mount Gravatt, QLD, Australia; ^3^Newcastle Law School, University of Newcastle, Callaghan, NSW, Australia

**Keywords:** simultaneous interpreting, remote interpreting, investigative interview, consecutive interpreting, interpreting mode

## Abstract

Remote interpreting via video-link is increasingly being employed in investigative interviews chiefly due to its apparent increased accessibility and efficiency. However, risks of miscommunication have been shown to be magnified in remote interpreting and empirical research specifically on video-link remote interpreting is in its infancy which greatly limits the evidence base available to inform and direct evidence-based policy and best practice, particularly in the identification of the optimal mode(s) of interpreting to be used, namely consecutive and simultaneous. Consecutive interpreting refers to a process in which the interpreter transfers short segments of speech from one language into the other as each person speaks in managed turn-taking, while simultaneous interpreting refers to the transfer of natural speech from one language into another in a concurrent manner without the need for speakers to segment their speech. This study provides novel empirical evidence by using eye tracking to compare the overt visual attention of interpreters working in a remote setting in which an English-speaking Interviewer interacts with a non-English-speaking Suspect in person, for whom interpretation is provided via video-link in real time. Using a within-subject design, we analyze eye-movement data from 28 professionally accredited interpreters who interpreted via video-link an investigative interview in which consecutive and simultaneous interpreting modes were counterbalanced. Taking interpreting performance into account, our results showed that, the consecutive mode yielded significantly less gaze time and therefore significantly less on-screen overt visual attention due to off-screen notetaking, an essential component of the consecutive interpreting mode. Relative to gaze time, the consecutive mode also resulted in significantly more and longer fixations and shifts of attention. Participants also allocated significantly more overt visual attention to the Interviewer than the Suspect, particularly in the consecutive mode. Furthermore, we found informative significant correlations between eye tracking measures and interpreting performance: accuracy, verbal rapport, and management. Finally, we found no significant differences between the three language pairs tested. We conclude with a discussion of limitations and the contributions of the study and an outline for future work on this topic of growing importance.

## Introduction

Interpreting services are used to enable interlingual communication when participants in a given interaction do not speak a common language (Russano et al., 2014). Traditional face-to-face interpreting services involve the interpreter being physically present in a given location, however, recent technological advances have seen remote interpreting services proliferate even before the COVID-19 pandemic, particularly in high-stakes legal settings where miscommunication can result in improper process and outcomes and to the inaccessibility of human rights and justice. Remote interpreting refers to a form of interpretation in which the interpreter is not physically present in the same location as the other participants but uses audio- or video-link instead. With its origins in the 1970s in Australia, remote interpreting via telephone then developed all over the world in many sectors, particular in public services, before developing into more sophisticated teleconferencing setups and eventually become the video-link software we commonly use today (see Kelly, 2008; Ozolins, 2011; Wakefield et al., 2014; Shaffer and Evans, 2018). This form of interpreting, particularly using video-link, is increasingly being employed worldwide in a variety of interpreting contexts, including legal, forensic, and investigative interactions, chiefly due to its accessibility and efficiency as it can potentially provide access to on-demand professional interpreting services regardless of physical location (e.g., Impli Project, 2012). The increasing availability and accessibility of videoconferencing solutions (e.g., Kelly, 2008; Licoppe and Veyrier, 2017) have resulted in remote interpreting becoming a viable alternative to face-to-face interpreting where an interpreter must physically travel to a given location, often at short notice. The issue of distance is particularly problematic for underrepresented language pairs in new, emerging, migrant, and minority languages and in advanced interpreting specializations (e.g., legal) where there is a further limitation to the availability of professionally accredited interpreters as practitioners are required to complete advanced qualification and professional accreditation testing that is above the general professional level (e.g., see National Accreditation Authority for Translation and Interpreters [NAATI], 2021).

The present study reports on a study of video-link remote interpreting collated as part of a large-scale project that aims to increase the knowledge and evidence base on the effects of remote interpreting in police interviews by examining the quality of interpreting in a controlled experimental investigative interview simulation. More specifically, the present study focuses on the examination of overt visual attention and interpreting performance of professional interpreters during consecutive and simultaneous interpreting modes in a remotely interpreted investigative interview. After a critical review of the current literature on consecutive and simultaneous modes (section “The Consecutive and Simultaneous Modes of Interpretin”) and the benefits and risks of remote interpreting (section “Benefits and Risks of Remote Interpreting *via* Video-Link”), we will then move to examine previous studies of visual attention in complex multimodal bi- and multilingual language processing tasks (section “Visual Attention in Multimodal Bi- and Multilingual Language Processing Tasks”), leading to a description of the study aims (section “Study Aims”) and a comprehensive description of the method, sampling, materials, and procedures used in this study (section “Materials and Methods”). We will then present our results (section “Results”) and discuss them within the context of the reviewed literature and wider professional practice (section “Discussion”). Finally, we conclude with a discussion of the contributions and limitations of the current study and link them to avenues of future research.

### The Consecutive and Simultaneous Modes of Interpreting

Face-to-face interpreting typically employs one of two modes, namely the consecutive mode and the simultaneous mode, which are typically associated with the nature of their physical surroundings and require activation of both source and target language, executive control, attentional focus, and coordination skills (Gile, 1995; Christoffels et al., 2006; Seeber, 2011). Consecutive interpreting refers to a process in which the interpreter is physically close to the speakers and transfers short segments of speech from one language into the other as each person speaks in managed turn-taking. The process continues back and forth between speakers and relies on all speakers explicitly segmenting their speech into manageable chunks of information and on the interpreter’s ability to correctly store and recall information in these chunks (Gile, 2009; Pöchhacker, 2011b; Diriker, 2012; Viezzi, 2012). Notetaking is a unique and essential part of the consecutive interpreting mode and involves the interpreter writing, editing, drawing, and reading notes so as to accurately record and use information before, during, and after the above speech segments. In court settings, an interpreter in the consecutive mode typically stands or sits next to the witness, whereas in legal interviews, including police settings, the interpreter usually sits equidistant from the two speakers, in a triangular position. The consecutive mode of interpreting is the most commonly used in domestic legal settings, including courts, tribunals, and interviews (e.g., Hale and Stern, 2011; Shaffer and Evans, 2018), and typically deals with scenarios in which two languages are being used.

Simultaneous interpreting refers to a process in which the interpreter transfers natural speech from one language into another in a concurrent manner without the need for speakers to segment their speech or pause and without the need for the interpreter to engage in notetaking activities (Christoffels et al., 2006; Gile, 2009; Pöchhacker, 2011a; Diriker, 2012; Seeber, 2012; Viezzi, 2012). In domestic legal settings, interpreters use the simultaneous mode without any equipment, interpreting in a whisper, often referred to as chuchotage. This requires the interpreter to sit very close to the accused in order to be heard. The lack of equipment also makes it difficult for interpreters to hear the speakers clearly, making this mode inconvenient, inefficient and not effective in delivering accurate interpreting (Hale et al., 2017). In contrast, in international settings, interpreters are typically not physically close to the speakers and employ a range of technologies, including headsets with microphones and headphones, so that the interpretation is delivered at almost the same time as the original utterance. This form of simultaneous interpreting is typically used in international conferences and international courts, e.g., the International Criminal Court, and tribunals in which many languages are being used and there is provision of the physical space required, e.g., purpose built interpreting booths, and the necessary hardware and software (Stern, 2012).

Consecutive and simultaneous modes of interpreting are well established and have been extensively used to overcome the barriers of language and accessibility. Switching between simultaneous and consecutive modes is commonplace in professional practice, in research (see Viezzi, 2012; Orlando, 2014; Braun, 2019; Stachowiak-Szymczak, 2019; Bartlomiejczyk and Stachowiak-Szymczak, 2021; Chen et al., 2021), and in professional certification testing (e.g., National Accreditation Authority for Translation and Interpreters [NAATI], 2021). Of course, unique advantages and disadvantages of each mode of interpreting have been identified though the overall picture in the literature has arguably been identified as somewhat inconsistent largely to due to a variety of research methods being used in different ways and limitations in being able to articulate, generalize and replicate studies (e.g., see Gile, 2001; Russell, 2002; Ko, 2006; Hale and Stern, 2011; Ozolins, 2011; Liang et al., 2017; Lv and Liang, 2019; Goodman-Delahunty et al., 2020), including in the specific context of legal interpreting (e.g., Berk-Seligson, 1999; Hale et al., 2017). From a professional practice perspective, the strengths and weaknesses of each mode need to be well understood in order to identify the optimal parameters in the provision of interpreting, particularly in the context of remote interpreting, which has been shown to “magnify known problems of interpreting” when compared to traditional face-to-face interpreting (Braun, 2011, p. 4).

Consecutive interpreting does not require equipment, and interpreters in this mode are far more numerous due to it being the standard mode in the profession and in the professional accreditation system (e.g., see National Accreditation Authority for Translation and Interpreters [NAATI], 2021). However, this mode requires significantly more resources as it takes approximately twice the amount of time of monolingual communication and simultaneous interpreting, and arguably limits the natural flow of communication given the need for the speakers to artificially stop and start after each segment of speech, the need for the interpreter to engage in notetaking activities, and the explicit management of turn-taking (Pöchhacker, 2011b; Ewens et al., 2017; Hale et al., 2017, 2019a). Within a legal context, these parameters have been shown to limit the efficacy of interview techniques (Powell et al., 2017), risk miscommunication between speakers (e.g., Licoppe et al., 2018), negatively affect witness credibility (Hale et al., 2017) and negatively affect jurors’ memory and concentration (Hale et al., 2017). Lastly, while the simultaneous mode offers more seamless and natural communication in approximately the same amount of time as the original speech, it typically requires more physical space and specialized equipment in comparison to the consecutive mode. Further, it requires interpreters to have advanced training and/or qualifications and professional accreditation (e.g., see National Accreditation Authority for Translation and Interpreters [NAATI], 2021) thereby greatly reducing the supply of interpreters who are proficient in this mode, particularly in legal settings, which disproportionally affects migrant and minority languages and geographical areas where practitioners are not available, e.g., outside of metropolitan areas. Arguably, both modes, in their traditional face-to-face delivery, are limited by the need for the interpreter to be physically present in the location of the speakers, a requirement that has long since been problematic when there is an urgent need for interpreting services, for those outside of urban areas and in inaccessible or sensitive locations (e.g., for legal reasons), and, most recently, for those affected by restrictions related to the COVID-19 pandemic.

### Benefits and Risks of Remote Interpreting *via* Video-Link

In contrast to the limitations of face-to-face interpreting, remote interpreting, like other language technologies (see Doherty, 2016), appears to offer a more accessible and efficient means of interlingual communication that could address many of the aforementioned physical limitations, particularly around the access to a greater number of language combinations and a larger pool of interpreters with specializations (e.g., legal). However, there are numerous inherent risks associated with remote interpreting which may have a detrimental impact on the quality of interpreting and thus impair and impede communication between parties. In legal, forensic, and investigative contexts, the consequences of miscommunication and barriers to communication can have detrimental effects and may happen unknowingly to speakers given the interpreter is typically the only person able to comprehend both of the languages being used. In other words, an English-speaking police officer has no way of knowing if any given utterance is being interpreted accurately and fluently to the suspect and vice versa. Of course, *post hoc* assessments of the recording or transcript can be carried out by another accredited interpreter or translator, but this is rarely requested and is typically confined to the courtroom proceedings, and not related activities such as investigative interviews that occur outside of the courtroom (e.g., see Hayes, 2009).

Given the complete reliance on the hardware and software required for remote interpreting, technical risks naturally arise when the hardware and/or software are not functioning optimally, which typically results in poor quality audio and/or video feeds, the inability to hear a speaker properly, temporal delays in the audio and/or video feed, and gaps and distortions in the audio. Furthermore, the relative ease of access to the hardware and software (e.g., a laptop with Zoom and access to the Internet) required to deliver remote interpreting may be associated with a lack of adequate training amongst interpreters and users of interpreting services, a lack of preparation given to interpreters (see Wong, 2020), and insufficient protocols to guide the interpreter and the other parties in using this novel paradigm. Any one of these risks, once again, can have known and unknown significant and detrimental impact on communication and amplify existing issues inherent in interpreting, including the respective issues attributed to the consecutive and simultaneous modes (e.g., Wadensjo, 1998; Rosenberg, 2007; Braun, 2011).

In sum, limited research exists to direct the informed usage of remote interpreting more generally and video-link in particular (cf. Ko, 2006; Vrij et al., 2019; Goodman-Delahunty et al., 2020), and while recent work has established the their efficacy across languages compared to face-to-face interpreting and audiolink remote interpreting (e.g., Hale et al., forthcoming), there is not yet sufficient evidence to ascertain the optimal mode of interpreting to be used for video-link remote interpreting given its unique attributes relative to other forms. As such, the current study aims to contribute to this now growing body of empirical evidence by investigating the overt visual attention of professional interpreters in consecutive and simultaneous interpreting modes using video-link software in a remotely interpreted investigative interview. It also aims to identify if and how measures of interpreting performance correlate with a range of established measures of overt visual attention. To our knowledge, this study is the first to use eye tracking to investigate the overt visual attention of interpreters in a comparison of these modes in a video-link remote interpreting context or in investigative police interviews.

### Visual Attention in Multimodal Bi- and Multilingual Language Processing Tasks

The Eye-Mind Hypothesis (Just and Carpenter, 1980) directs our approach to investigating visual attention during the interpreting task given it is a multimodal and bilingual language processing task (Christoffels and De Groot, 2005; Doherty et al., 2010; Doherty and O’Brien, 2014; Doherty, 2020). The hypothesis is a core concept of the eye tracking methods in language processing research which posits a relatively immediate and direct relationship between eye movements and their fixations and what is being processed by the brain (Just and Carpenter, 1980; Liversedge et al., 2011). The hypothesis is operationalized in a visual attention system in which cognitive operations distinguish between, and filter, relevant and irrelevant visual information in order to efficiently process relevant information in a system with a limited capacity (Carrasco, 2011). In such systems, visual attention can be allocated in a top-down and bottom-up manner (see Buschman and Miller, 2007) in which both overt and covert visual attention is possible. Broadly speaking, top-down refers to task and context demands in which we actively attend to a given stimulus for a predefined reason, e.g., to follow an interviewer’s eye gaze and hands as they speak with a suspect, while bottom-up denotes an automatic attraction of visual attention caused by the stimuli itself, e.g., shifting our overt visual attention to focus on a sudden or unexpected movement.

There is an important distinction to be made between overt and covert visual attention (see Rai and Le Callet, 2018): while overt visual attention denotes the physiological act of the eye fixating on a stimulus, covert visual attention can precede, overlap, and succeed these fixations. The two are complementary and help us to navigate, process, and respond to complex and dynamic visual stimuli in our physical environment, for example, using covert visual attention to monitor a visual scene on the computer screen and then switching to overt visual attention to direct our eyes to fixate on a person appearing on the screen. Studies of covert and overt visual attention have consistently shown that cognitive processing, related and unrelated to the presented stimuli, can indeed occur outside of overt visual attention (e.g., Posner, 1980; Hunt and Kingstone, 2003; Irwin, 2004; Wright and Ward, 2008; Rai and Le Callet, 2018).

Experimental design can be used to avoid confounds in the attribution and interpretation of visual attention in order to substantiate the argument for a link between fixation-based eye movements and the cognitive processing isolated by the experiment (Irwin, 2004; Duchowski, 2007), even in highly ecologically valid naturalistic experiments such as ours. The presence of predefined target stimuli in defined areas, the designation of specific tasks, and the specification of spatiotemporal areas of interest are the most typical procedures used to substantiate the link between target stimuli and their processing and resultant observable behaviors. We have therefore incorporated these parameters in the design of the current study and while overt and covert visual attention are closely interlinked (see Rai and Le Callet, 2018), the focus of the current study is on overt visual attention given our usage of established eye tracking measures used in language processing, further detailed as follows.

Eye movements have become a widespread measure for monolingual and bilingual spoken language processing research (e.g., Spivey and Marian, 1999; Ju and Luce, 2004), particularly in their operationalization as part of the Visual World paradigm in which comprehension and production of language have been consistently and closely linked to eye movements (see Tanenhaus et al., 2000; Griffin, 2004b; Tanenhaus, 2007; Heuttig et al., 2011; Ferreira and Rehrig, 2019), including eye movement features employed in a concurrent, anticipatory, or subsequent manner, e.g., the “eye-voice span” (Levin and Buckler-Addis, 1979). In the words of Magnuson et al. (1999, p. 331) eye movements are “closely time-locked to the unfolding speech signal.” Of direct relevance here are the growing bodies of evidence showing a tight link between eye movements and the language processing tasks employed in interpreting, including naming (Meyer et al., 1998), planning and formulation (Griffin and Bock, 2000; Bock et al., 2003; Brown-Schmidt and Tanenhaus, 2006; Esaulova et al., 2019), coordination and perspective in conversation (Keysar et al., 2000; Hanna and Tanenhaus, 2004; Brown-Schmidt et al., 2005), and the processing of disfluencies (Arnold et al., 2004; Griffin, 2004a; Pistono and Hartsuiker, 2021). These studies have consistently identified and corroborated a strong link between eye movements at critical stages of comprehension and production processes, e.g., memory (e.g., Richardson and Spivey, 2000; Altmann and Kamide, 2004), phonology (Tanenhaus et al., 1995), semantics (e.g., Chernov, 1979; Dahan et al., 2001), syntax (e.g., Eberhard et al., 1995), and discourse (e.g., Altmann and Kamide, 2004). In sum, it is well established that more and longer fixations are associated with longer and more effortful cognitive processing (Pannasch et al., 2001; Holmqvist et al., 2015) noting that the location of fixation duration generally corresponds to the central point of vision although visual processing incorporates a much larger area (Irwin, 2004, p. 107).

Further, within the literature on interpreting, the links between eye movements and language comprehension and production are relatively less established but indeed present and notable (see Stachowiak-Szymczak, 2019), where several studies have expanded on the lines of research described above. A recent review by Tiselius and Sneed (2020) provides considerable interpreting-specific evidence that substantiates the link between language comprehension and production in a variety of interpreting tasks and contexts, including the use of visual stimuli to support language processing (e.g., Seeber, 2012; Tiselius and Sneed, 2020), the resolution of incongruency (Stachowiak, 2017), the importance of eye movements in enabling interpreted conversations (Krystallidou, 2014; Vranjes and Brône, 2020), monitoring turn-taking (Vertegaal et al., 2003; Bot, 2005; Mason, 2012; Davitti, 2019), and indicating mis/understanding (Mason, 2012), and the unique gaze patterns identified in backchanneling (Vranjes et al., 2018) and note-taking (Chen et al., 2021). Similarly, contemporary models of interpreting incorporate such complexity and the multi-tasking nature of language comprehension, production, and coordination associated with language processing in interpreting (e.g., Chernov, 1994; Gile, 2009; Seeber, 2017), where Gile (2009, p. 166), in particular, identifies the need for the optimization of “efforts” between “listening” and “production,” in which too much capacity being used in one results in diminished capacity in the other, and potential interference may occur and result in overload and consequent poor performance (Seeber, 2011; Seeber, 2013) as the interpreter walks the “tightrope” of cognitive saturation (Gile, 1999).

Several eye-tracking measures are relevant to bilingual language (see Liversedge et al., 2011). Doherty and Kruger (2018) critically reviewed and categorized eye-tracking measures for use in multimodal and multilingual language processing research and distinguished between primary and secondary eye-tracking measures where the former refers to raw values derived directly from eye movements and the latter refers to measures that combine two or more primary measures to be constructed. The distinction is useful to our purposes here and allows us to identify that eye-tracking studies of bilingual language processing make use of both primary and secondary measures depending on the research question and design. As our participants are engaged in interpreting tasks in the current study, we rely on primary measures of gaze time: the duration of time of overt visual attention on screen as measured by fixations (Holmqvist et al., 2015, p. 389); fixation count: the number of times the eyes is relatively still in a given position (Holmqvist et al., 2015, p. 412), fixation duration: the temporal duration of a given fixation (Holmqvist et al., 2015, p. 377), and the secondary measure of shifts of overt visual attention: a fixation in a different defined area of interest to the previous fixation.

### Study Aims

To address the prevailing gaps in the interpreting literature and in evidence-based best professional practice, the current study aims to use established eye tracking measures to examine and compare the overt visual attention of professional interpreters in consecutive and simultaneous interpreting modes using video-link in a remotely interpreted live investigative interview. It also aims to explore the relationship between established measures of overt visual attention used in language processing research and established measures of interpreting performance. Informed by the above critical review of relevant literature, we pose the following research questions and corresponding hypotheses.

1. Is there a difference in interpreters’ overt visual attention between consecutive and simultaneous modes of remote interpreting?
2. Is there a correlation between overt visual attention and interpreting performance?
3. Are there significant differences between language combinations?

Given that interpreters engage in off-screen notetaking activities in the consecutive mode, we hypothesized that overt visual attention would be relatively greater in the consecutive mode than the simultaneous mode as reflected in longer overall gaze time (Hypothesis 1a). We also hypothesized the consecutive mode to have a higher fixation count (Hypothesis 1b), longer mean fixation duration (Hypothesis 1c) and more shifts of overt visual attention (Hypothesis 1d). Further, we hypothesized that there would be a correlation between the above measures of overt visual attention and established measures of interpreting performance (Hypothesis 2). Finally, we aimed to include a variety of language combinations to extend the generalizability of our findings, English < > Arabic, English < > Chinese, and English < > Spanish, but we do not anticipate there to be differences between them (Hypothesis 3).

## Materials and Methods

### Participants

We used convenience sampling to recruit 28 interpreters, 20 identified as female and eight as male, aged between 23 and 73 years (*M* = 44.25, *SD* = 14.07) in three languages: Arabic (*n* = 5) Mandarin Chinese (*n* = 13) and Spanish (*n* = 10). A gender balance was not possible as the above ratio is typical of the interpreting profession. The sample is in line with comparable studies in interpreting research in which eye tracking is employed (see Stachowiak-Szymczak, 2019; Tiselius and Sneed, 2020). All participants had a professional accreditation and/or formal qualification in interpreting (24 had both) and had specific experience with remote interpreting. Recruitment was carried out by electronic mailing lists to professional networks and associations. All participants were recruited by the authors’ posting to electronic mailing lists linked to professional networks and associations in accordance with the institutional guidelines on human research participation at the authors’ institutions. The participants provided their written informed consent to participate in this study and were remunerated for their time in accordance with institutional ethics guidelines. The study was reviewed and approved by the Federal Bureau of Investigation Institutional Review Board (378-16), the Charles Sturt University Human Research Ethics Committee (H16164), and the University of New South Wales Human Research Ethics Committee (H16164).

### Materials

We designed a purposefully scripted interview of approximately 2,000 words in English based on previous real-life investigative interviews and the established standards of professional interpretation (see Liu and Chiu, 2009; Hale et al., 2019b). The script was reviewed by experienced police interviewers to ensure their plausibility and translated into Arabic, Mandarin Chinese, and Spanish by professional translators with accreditation from the National Accreditation Authority for Translators and Interpreters and a post-graduate degree in translation from an Australian university. The translated scripts were checked and edited in discussion with a second translator with the same credentials (scripts can be found under Supplementary Material).

The script was enacted by professionally trained actors to ensure consistent performance and ecological validity for all participants regardless of language combination. The English component was performed by the same Interviewer across all languages, and the target language component had the translated version in the respective language and was performed by three different actors who were native speakers of each of the three languages and played the role of the Suspect.

Participants’ interpreting performance was assessed using a transcript of their interpretation which was coded by two trained coders in each language each with professional accreditation in interpreting and specific experience in coding interpreting data. Each transcript was assessed for interpreting performance using a set of validated weighted criteria widely used in professional interpreting examinations and in previous research (see Hale et al., 2019b), namely: accuracy of propositional content, accuracy of style, maintenance of verbal rapport markers, use of interpreting protocols, and use of legal discourse and terminology. The assessment criteria were weighted with each criterion ranging from a minimum of zero points and a maximum of ten points: a total of 100% overall. The mean inter-rater reliability score was acceptable (α = 0.85), and where agreement fell below α = 0.7, a third independent rater assessed the transcripts. The final assessment score was the mean value between the two coders, or, if a third coder was involved, the mean between the results from the two raters who achieved the highest interrater reliability score.

### Procedure

The interviews were all held at a professional interview facility in Sydney, Australia. The facility had a standard office room for the interview to take place between the English-speaking Interviewer and non-English-speaking Suspect, and a separate room for the interpreter to view the interview on a computer screen to which a non-invasive eye tracker was equipped, as detailed below. After providing informed consent, participants completed a controlled experimental laboratory study in the form of a 30-min realistic simulated investigative interview by an English-speaking *Interviewer* of a *Suspect* who does not speak English, but speaks Arabic, Chinese, or Spanish depending on the language pair of the interpreter. The English-speaking Interviewer was constant across all languages. To enable counterbalancing, the order of interpreting mode was systematically varied within each interview session so that participants completed one half of the interview in the consecutive mode and the other half in the simultaneous mode. A set point in the text was used to switch between modes and the exact time of switching was recorded for each participant given the time varied slightly owning to the individual pace of each interpreter. The interviewers were instructed on the task at hand, including the need to switch modes at a particular point. In order to minimize or avoid possible spill-over effects, each interpreter had sufficient time after switching to begin in the new mode. This type of switching between simultaneous and consecutive modes is common in professional practice and in research examining modes (see Diriker, 2012; Viezzi, 2012; Orlando, 2014; Braun, 2019; Stachowiak-Szymczak, 2019; Bartlomiejczyk and Stachowiak-Szymczak, 2021; Chen et al., 2021), and aligns with the professional certification (see National Accreditation Authority for Translation and Interpreters [NAATI], 2021) and specific professional experience of the targeted sample for the current study. Each half of the interview corresponded to 1,000 words of the 2,000-word script, where word counts are calculated using the English source text. We recorded precise time coding for when each participant switched between modes. The interpreters viewed the interview in full-screen mode via Zoom, a remote video conference software program often used for video-link remote interpreting (Zoom, 2001).

### Eye Tracking

**FIGURE 1:**
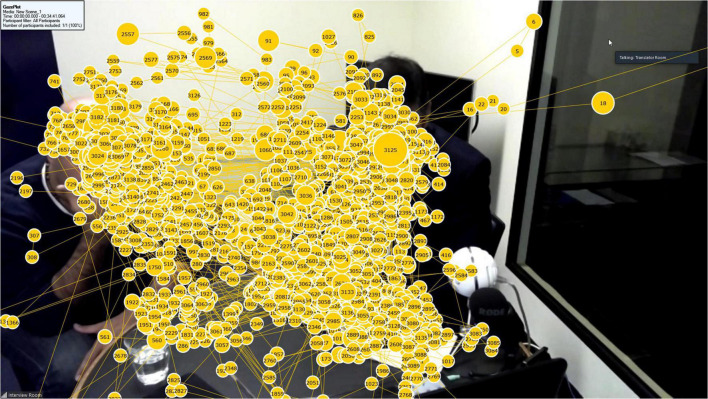
Example of gaze data for a participant in the consecutive mode.

**FIGURE 2:**
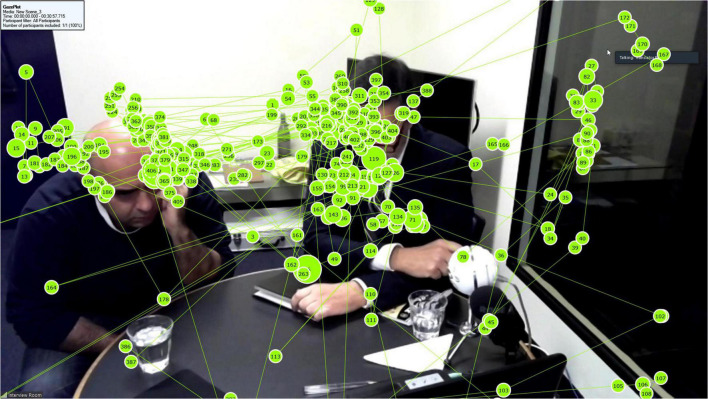
Example of gaze data for a participant in the simultaneous mode.

We used a Tobii Pro X2-60 screen-based eye tracker to capture eye movements. The device has a temporal resolution of 60 Hz and binocular accuracy between 0.4 and 1.2 degrees, thus allowing for a limited range of free head movement required for the study. Its precision is rated at 0.32 degrees. We used the screen record function of Tobii Pro Studio (version 3.4.1) to record participants’ eye movements as they observed the interview via the full screen Zoom window. Figures 1, 2 show typical examples of the recorded gaze data overlaid onto the interview scene, that is, the scene presented to the participants in the consecutive mode (Figure 1) and the simultaneous mode (Figure 2). Fixation counts appear in order of occurrence and their size corresponds to the temporal duration of each fixation, i.e., longer fixations appear as larger circles.

We calibrated for each participant in each recording using a 9-point calibration to maximize the likelihood of usable data. We carried out a manual visual inspection on each recording before and after applying the Tobii I-VT fixation filter to filter raw data (Olsen, 2012) and rejected low quality data below comparable thresholds for bilingual language processing tasks (see Doherty, 2012, 2018).

Lastly, we created an area of interest (AOI) around the Interviewer, and the Suspect. While the Interviewer and Suspect generally remained in their vertical half of the screen, the AOIs were dynamic to account for their changes in movement on the screen and over the duration of each recording. Finally, we exported all data from Tobii Pro Studio to R (version 3.5.1) for post processing and analysis due to the large size of the datasets.

## Results

### Statistical Testing

We performed a series of descriptive and inferential statistical testing on each of the eye tracking measures described below. For each dependent variable and each category of the independent variable, e removed outliers of more than two standard deviations from the mean and by visual inspection of individual boxplots for each dependent variable: *gaze time* (1 data point in the positive direction, 2 negative; 5.36%), *fixation count* (2 data points in the positive direction, 0 negative; 3.57%), *mean fixation duration* (1 data points in the positive direction, 2 negative; 5.36%), and *shifts of overt visual attention* (0 data point in the positive direction, 1 negative; 1.79%) – overall, data loss well within the norm for similar studies in interpreting and translation tasks, where values of up to 10% have been found for lab-based tasks, and up to 30% for field tasks (e.g., see Saldanha and O’Brien, 2014). Normality, homogeneity of variances, and sphericity were assessed using Shapiro-Wilk’s test, Levene’s test, and Mauchly’s test, respectively. Bonferroni’s adjustment was used to account for multiple comparisons. We employed repeated-measures ANOVAs, as detailed in section “Difference between Modes on Eye Tracking Measures,” to test for differences between modes with covariates of language combination and interpreting performance. We then removed language as a covariate as it had no significance in any of the following analyses, a result that aligns with previous findings of no differences between languages, see section “Discussion”). We then use the aforementioned AOIs to examine each of the eye tracking measures relative to the Interviewer and the Suspect. Finally, we conducted correlational analyses to identify the relationships between each of the eye tracking measures and the criteria used to measure interpreting performance.

### Difference Between Modes on Eye Tracking Measures

Summary descriptive statistics for each of the eye tracking measures can be found in Table 1: *gaze time*, *fixation count*, *mean fixation duration*, and *shifts of overt visual attention*. A repeated-measures ANOVA identified a significant difference between *modes* on total *gaze time*, where the *consecutive mode* was significantly lower than the *simultaneous mode*, *F*(1, 54) = 173.68, *p* < 0.001, ω^2^ = 0.12, *d* = 0.81. The lower *gaze time* in the *consecutive mode* can be accounted for by the participants having to look away from the screen to engage in note-taking activities (writing, editing, drawing, and reading, see Chen, 2016; Chen et al., 2021).

**TABLE 1:** Summary descriptive statistics for eye tracking measures between *modes.*

Measure	*Mode*			
	*Consecutive*		*Simultaneous*	
	*Mean*	*SD*	*Mean*	*SD*
*Gaze time* (seconds)	1096.65	54.42	1563.25	179.27
*Fixation count* (raw count)	1125.24	202.54	981.52	46.24
*Mean fixation duration* (milliseconds)	402.21	6.10	368.51	9.99
*Shifts of overt visual attention* (raw count)	658.28	15.97	541.35	12.10

We conducted a repeated-measures ANOVA which showed a significant difference between *modes* on mean *fixation count*, where the *consecutive mode* was higher than the *simultaneous mode*, *F*(1, 54) = 13.40, *p* < 0.001, ω^2^ = 0.11, *d* = 0.62. We also found a significant difference between *modes* on *mean fixation duration*, where the *consecutive mode* was higher than the *simultaneous mode*, *F*(1, 54) = 231.30, *p* < 0.001, ω^2^ = 0.07, *d* = 0.59. Lastly, we found a significant difference between *modes* on the number of *shifts of overt visual attention* between AOIs, where the *consecutive mode* was higher than the *simultaneous mode*, *F*(1, 54) = 953.76, *p* < 0.001, ω^2^ = 0.05, *d* = 0.57.

To further explore the distribution of overt visual attention on the screen during the interpreting tasks. we isolated each of the eye tracking measures for each AOI to examine the distribution of overt visual attention on the *Full Scene* and its components: the *Interviewer* and the *Suspect* (see Table 2). A repeated-measures ANOVA identified significantly greater allocation of overt visual attention attributed to the *Interviewer* with longer *gaze time* [*F*(1, 54) = 132.22, *p* < 0.001, ω^2^ = 0.12, *d* = 0.71], higher *fixation count* [*F*(1, 54) = 11.34, *p* < 0.01, ω^2^ = 0.08, *d* = 0.66], longer *mean fixation duration* [*F*(1, 54) = 201.93, *p* < 0.001, ω^2^ = 0.07, *d* = 0.72], and more *shifts of overt visual attention* [*F*(1, 54) = 453.61, *p* < 0.001, ω^2^ = 0.07, *d* = 0.70] all of which were significantly higher for the *Interviewer*. A repeated-measures ANOVA which also showed the *consecutive mode* to result in significantly shorter *gaze time* [*F*(1, 54) = 112.20, *p* < 0.001, ω^2^ = 0.09, *d* = 0.86], higher *fixation count* [*F*(1, 54) = 13.81, *p* < 0.01, ω^2^ = 0.11, *d* = 0.77] with longer *mean fixation duration* [*F*(1, 54) = 191.23, *p* < 0.01, ω^2^ = 0.07, *d* = 0.72], and more *shifts of overt visual attention* [*F*(1, 54) = 313.15, *p* < 0.01, ω^2^ = 0.06, *d* = 0.67].

**TABLE 2:** Summary descriptive statistics for eye tracking measures across areas of interest.

Measure	Area of Interest					
	*Full scene*		*Interviewer*		*Suspect*	
	Mean	*SD*	Mean	*SD*	Mean	*SD*
*Gaze time* (seconds)	1327.55	207.81	727.54	25.88	600.00	32.45
*Fixation count* (raw count)	1291.88	98.37	821.12	21.11	570.88	28.37
*Mean fixation duration* (milliseconds)	391.23	12.89	411.52	17.65	370.22	19.98
*Shifts of overt visual attention* (raw count)	911.22	26.71	551.71	27.88	359.51	21.35

Finally, we examined the temporal aspects of the latter two measures splitting the data from each recording into four segments, i.e., the *consecutive mode* had two halves of equal duration, and the *simultaneous mode* had two halves of equal duration. A repeated-measures ANOVA revealed a significant main effect of *time* [*F*(1, 107) = 114.12, *p* < 0.001, ω^2^ = 0.13, *d* = 0.89] with a significant interaction between *time* and *mode* [*F*(1, 107) = 101.86, *p* < 0.001, ω^2^ = 0.10, *d* = 0.55], where *fixation count* increased in each block of *time* in both *modes* (*p* < 0.001, *d* = 0.84) and was significantly higher in the *consecutive mode* (*p* < 0.01, ω^2^ = 0.08, *d* = 0.72), and *mean fixation duration* also increased in each block of *time* in both modes (*p* < 0.001, ω^2^ = 0.08, *d* = 0.88) with the *consecutive mode* significantly higher than the *simultaneous mode* (*p* < 0.01, ω^2^ = 0.07, *d* = 0.68).

### Relationship Between Eye Tracking Measures and Interpreting Performance

The correlational analyses for the eye tracking measures and interpreting performance are detailed in Table 3. Given the multidimensional nature of interpreting performance (see section “Materials”) and the usage of multiple eye tracking measures in the above analyses, we aimed to explore separate, individual correlations, which identified significant correlations between each of the eye tracking measures of *gaze time, fixation count, mean fixation duration*, and *shifts of overt visual attention.* As the primary aim of these correlational analyses is indeed to explore the relationship between the above eye tracking and interpreting performance, we report individual correlations (Pearson’s *r*) with the Bonferroni correction for multiple comparisons.

**TABLE 3:** Pearson’s *r* correlation coefficients between eye tracking measures and interpreting performance.

	Eye tracking measures				Interpreting performance		
		
	*Gaze time*	*Fixation count*	*Mean Fixation duration*	*Shifts of overt visual attention*	*Accuracy*	*Rapport*	*Management*
*Gaze time*		−0.41**	−0.79**	−0.84**	0.28*	0.25*	0.25*
*Fixation count*			0.36*	0.40*	0.25*	0.21*	0.32*
*Mean Fixation duration*				0.87**	0.23*	0.21*	0.29*
*Shifts of overt visual attention*					0.26*	0.29*	0.38*
*Accuracy*						0.45*	0.77*
*Rapport*							0.49*

The Pearson’s correlation coefficient (r) is displayed after Bonferroni correction with significance levels of* for p < 0.05, and **p < 0.01.

Such significant moderate to very strong correlations between these eye tracking measures (*gaze time, fixation count, mean fixation duration*, and *shifts of overt visual attention*) are to be expected given the basis and calculation of the individual measures (see section “Visual Attention in Multimodal Bi- and Multilingual Language Processing Tasks”). In other words, the longer the time spent gazing on screen, the more likely fixations and shifts of overt visual attention are to take place. Further, except for *shifts of over visual attention*, the remaining eye tracking measures also correlated significantly with each measure of interpreting performance, where *accuracy* had weak correlations with each eye tracking measure, as did *rapport* and *management*. Through the effects are generally quite weak, such a set of results indicates that the longer that the participants fixated on the speakers, as opposed to looking away from speakers to engage in note-taking activities, the more accurate their interpretation. Further, it appears that the more the interpreters shifted their overt visual attention between speakers, as opposed to looking away from speakers to engage in note-taking activities, the better the *rapport* and *interaction management* scores. While it is indeed informative and adds validity to our measures and materials, it is also unsurprising to see the significant strong and medium correlations between the sub-components of interpreting preference given they have already been previously validated as a standardized measurement (see Hale et al., 2019b). Due to the limited statistical power of the data available for mode-specific correlations, we have not included them as they do not add any meaningful data and align with the above findings across both modes.

## Discussion

It is evident that remote interpreting via video-link is increasingly being employed (e.g., Kelly, 2008), particularly during the COVID-19 pandemic, due to its apparent increased accessibility, effectiveness, and efficiency, particularly in legal settings. However, our review of literature has clearly identified that significant and impactful risks miscommunication have been shown to be introduced and even amplified in remote interpreting and empirical research specifically on video-link remote interpreting is in its infancy which greatly limits the evidence base available to inform and direct evidence-based policy and best practice, particularly in the identification of the optimal mode(s) of interpreting and the interpreters’ processing of the complex and dynamic multimodal and bilingual language stimuli inherent in the video stimuli. As such, the current study aimed to examine and compare the overt visual attention in consecutive and simultaneous modes in a remote-interpreted investigative interview (RQ1) vis-à-vis established measures of interpreting performance (RQ2) and across combinations of major language pairs (RQ3). As hypothesized, the consecutive mode resulted in significantly shorter total *gaze time* with a large effect size (Hypothesis 1a) due to interpreters in this mode engaging in extensive note-taking activities. Noting that within-subjects ANOVA has a higher statistical power than a between-subjects ANOVA (see Lakens, 2013). Similarly, we found the *consecutive mode* to have higher *fixation count* (Hypothesis 1b), longer *mean fixation duration* (Hypothesis 1c), and more *shifts of overt visual attention* (Hypothesis 1d) than the *simultaneous mode*; each with medium effect sizes. We also found that participants consistently attributed more overt visual attention to the *Interviewer* than the *Suspect*, particularly in the *simultaneous mode*, with large to medium effect sizes, respectively.

Further, the finding, and medium effect sizes, of more and longer fixations in the second segment of each mode clearly indicates the importance of task time in remote interpreting load, with the *consecutive mode* again being significantly more cognitively demanding than the *simultaneous mode*. Such a result is unsurprising given cumulative load has been previously identified in a variety of interpreting tasks (e.g., Gile, 2009; Seeber, 2012, 2017; Chen, 2017; Defrancq and Plevoets, 2018, see also Kruger and Doherty, 2016). Lastly, we found consistent significant correlations between the above eye tracking measures, except for *shifts of visual attention*, and each measure of interpreting performance (Hypothesis 2), particularly for *accuracy*, where more overt visual attention on the speakers was associated with more accurate interpretation. As expected, we did not find any differences between languages on any measure (Hypothesis 3) noting our sample was uneven in this regard.

Based on our findings, we argue that off-screen note-taking activities can account for the identified consistent significant differences between *modes* whereby the *consecutive mode* resulted in more fixations and longer *mean fixation durations* relative to *gaze time*. As such, our results largely indicate a need for the interpreter in this mode to continually switch their overt visual attention off and on screen and to engage in a reorientation and resultant spatiotemporal integration (see Doherty, 2020) after each switch due to the complex and dynamic nature of the video-link stimuli. In other words, although participants engaged in note taking to facilitate the interpreting task, they may have had to catch up on the visual events that they missed, at least visually, while looking off screen. This argument is supported by the *consecutive mode* resulting in a greater number of *shifts of overt visual attention*. Given the link between measures of overt visual attention, particularly *fixation count* and *mean fixation duration*, our results also suggest that the *consecutive mode* is more cognitively demanding in a video-link remote interpreting context (cf. Pannasch et al., 2001; Holmqvist et al., 2015).

The results of the *Interviewer* attracting more overt visual attention than the *Suspect* is also of importance, particularly as it was more apparent in the *consecutive mode*. This may be explained by the power dynamic (see Mason and Ren, 2012) between the *Interviewer* and *Suspect* and/or the need to allocate overt visual attention to the interview in order to improve comprehension, for instance, by attending to non-verbal information, ambiguous information, or other information that they may have missed while engaging in off-screen note-taking activities. It may also be related to the more difficult legal language used by *Interviewer*, as compared to the more colloquial language of the *Suspect*. Further work is clearly needed to better examine any casual relationships in such a complex social, cognitive, linguistic, and technological process, particularly in legal contexts (Christensen, 2008).

### Limitations

The limitations of the current study are its relatively small sample size and uneven numbers across languages caused directly by the limited number of suitable interpreters available to the project despite extensive recruitment. Arguably, the current study has sufficient ecological validity given its authentic design (task, materials, participants) in which an authentic interpreting task is examined in a systematic, counterbalanced, and controlled manner using a within-subjects design. More than one interpreting task could have been used for each *mode*, i.e., a counterbalance of one full interview in *consecutive mode* and one full interview in *simultaneous mode*, but this design would have at least doubled the time and cost required for each interpreter, as a break would be needed given one interview corresponds to typical professional practice (see Hale et al., 2019b) and may also introduce a confound of comparability between tasks.

Given the impact that note-taking activities had on the interpreters in the consecutive mode, one could argue its exclusion to enable a more one-to-one comparison with the simultaneous mode, however, to identify a causal relationship, future work would have to remove notetaking from the equation, but such a modification would then completely compromise the ecological validity of the mode given how central notetaking is to consecutive interpreting (see Chen, 2016). Future work could explore the relationship between visual attention, cognitive load, and note-taking given recent studies have shown note-taking to be cognitively demanding with the potential to reduce interpreting-related cognitive effort (Stachowiak-Szymczak, 2019; Chen et al., 2021) which may or may not be realized due to cognitive capacity (cf Gile, 2009; Seeber, 2017).

Further, our profiling of participants could be improved upon by employing established quantitative measures of working memory capacity and language proficiency as these variables have been shown to have a significant and consistent impact on language performance in multimodal tasks. Further to this, a comparison of interpreting modes in face-to-face and remote settings would improve the generalizability of our findings and further inform evidence-based policy and best practice on this topic of growing importance given its potential to increase or decrease access to justice, procedural fairness, and appropriate outcomes. Lastly, the current study did not investigate the underlying cognitive processing behind the allocation of overt visual attention, a study of such processing and the associated cognitive load imposed by each mode of interpreting would further substantiate the results reported here, especially with regard to interpreting performance over time in remote settings and to separate eye movements vis-à-vis language comprehension and language production, as has been carried out in previous, largely monolingual language processing research (as reviewed in section “Visual Attention in Multimodal Bi- and Multilingual Language Processing Tasks”). We hope that the findings of the current study and our own future work can support future work to address these remaining deficits on this topic.

### Contributions

To our knowledge, this is the first study of remote interpreting in which eye tracking is used to investigate the overt visual attention of interpreters in consecutive and simultaneous modes of interpreting. As such there is a limited capacity to directly compare results, e.g., effect sizes and correlation coefficients, with comparable literature in interpreting owing to “paucity” of empirical work identified by Stachowiak-Szymczak (2019). Using a combination of online and offline methods to analyze process and product, our findings add to a limited body of empirical evidence on the efficacy of interpreting modes, in which we show further advantages in terms of overt visual attention and performance of the simultaneous mode over consecutive due to interpreters in the latter mode allocating their visual attention on-screen and off-screen to engage in notetaking activities which appears to cause a disruption to the flow of visual input on the screen and an increased need to reorientate and reintegration visual attention after each off-screen switch resulting in more and longer fixations, which have been extensively identified in the language processing literature as being associated with more effortful cognitive processing (Pannasch et al., 2001; Holmqvist et al., 2015) particularly in the context of speech processing given the “time-locked” nature of eye movements and speech (Magnuson et al., 1999, p. 331). Our findings may not be surprising given the previous findings of eye tracking in interpreting (section “Visual Attention in Multimodal Bi- and Multilingual Language Processing Tasks”), though not numerous (Stachowiak-Szymczak, 2019; Tiselius and Sneed, 2020), show the critical importance of visual attention in face-to-face and remote interpreting tasks, including enhanced language processing (e.g., Seeber, 2012; Tiselius and Sneed, 2020), resolution of incongruent input (Stachowiak, 2017), coordination and interpreted conversations monitoring turn-taking (Vertegaal et al., 2003; Bot, 2005; Mason, 2012; Davitti, 2019; Krystallidou, 2014; Vranjes and Brône, 2020), and signaling issues in comprehension (Mason, 2012).

Predictably, our findings further substantiate the link between overt visual attention and language processing (as reviewed in section “Visual Attention in Multimodal Bi- and Multilingual Language Processing Tasks”), in this case multimodal bilingual language processing. We saw, for instance, how interpreting performance consistently linked to overt visual attention, where gaze time had the strongest correlation with interpreting accuracy and shifts of overt visual attention correlated strongest with rapport and management. It is also evident from our findings that both modes of interpreting resulted in overt visual attention being attended to the Interviewer more than the Suspect. These findings, coupled with the high ecological validity of the study in which an authentic interpreting task is examined across languages in a systematic and controlled manner, contribute to the knowledge and evidence base of multiple disciplines and areas of practice and indeed to much needed policy for informed and evidence-based best practice in remote interpreting scenarios (see Braun, 2011), particularly in legal contexts (see Hale, 2020).

Moreover, the analyses presented here naturally focussed on a high-level comparison between modes owing to the posed research questions, yet future work from this dataset could explore in more fine-grained analyses, e.g., at the level of segments or turns, links between eye movements and components of interpreting, namely comprehension, production, and coordination. Given the depth of empirical evidence surrounding these aspects of monolingual language processing (as reviewed in section “Visual Attention in Multimodal Bi- and Multilingual Language Processing Tasks”) it would be used to explicitly base this work in the Visual World paradigm (see Tanenhaus, 2007; Ferreira and Rehrig, 2019) and form a more explicit link between such work and contemporary models of interpreting given the overlap (e.g., Chernov, 1994; Gile, 2009; Seeber, 2017).

In terms of professional practice, these findings highlight the benefits of the simultaneous interpreting mode over the consecutive mode. They also emphasize the importance of interpreters in remote settings focussing their visual attention on the speaker (see Griffin, 2004a) to gather as much verbal and non-verbal information as possible in each turn to increase the likelihood of an accurate interpretation despite the urge to avert their gaze to reduce the cognitive burden of incoming visual information (cf. Glenberg et al., 1998; Doherty, 2020). Further to this, the ability to shift visual attention between speakers appears to be of great importance to interpreters so that they appropriately manage the interact and ensure they build and sustain rapport with each speaker involved in the interaction. Finally, the presence of increased load in the second half of each mode highlights the importance of adequate breaks for interpreters in such a demanding task, particularly in consecutive mode.

## Data Availability Statement

The datasets presented in this study can be found in online repositories. The names of the repository/repositories and accession number(s) can be found below: The data used for the statistical analyses that support the findings of this study are available from doi: 10.13140/RG.2.2.30324.42882.

## Ethics Statement

The studies involving human participants were reviewed and approved by the Federal Bureau of Investigation Institutional Review Board (378-16), the Charles Sturt University Human Research Ethics Committee (H16164), and the University of New South Wales Human Research Ethics Committee (H16164). The patients/participants provided their written informed consent to participate in this study. Written informed consent was obtained from the individual(s) for the publication of any potentially identifiable images or data included in this article.

## Author Contributions

All authors contributed to interpretation of analyses and approved the final version of the manuscript.

## Conflict of Interest

The authors declare that the research was conducted in the absence of any commercial or financial relationships that could be construed as a potential conflict of interest.

## Publisher’s Note

All claims expressed in this article are solely those of the authors and do not necessarily represent those of their affiliated organizations, or those of the publisher, the editors and the reviewers. Any product that may be evaluated in this article, or claim that may be made by its manufacturer, is not guaranteed or endorsed by the publisher.
