# Supplementary material for: An Eye-Movement Analysis of Overt Visual Attention During Consecutive and Simultaneous Interpreting Modes in a Remotely Interpreted Investigative Interview
Source: Front Psychol. 2022 Mar 25;13:764460. doi: 10.3389/fpsyg.2022.764460 (PMC8992695; doi:10.3389/fpsyg.2022.764460)
Supplement: Supplementary file 1 [file Data_Sheet_1.docx]

Police interview

POLICE: (To interpreter) Hello Mr/Madam Interpreter. I’m glad you were able to come on time. We’ll be interviewing this man who we understand speaks only Mandarin and can’t speak English, so we need you to interpret for us. Can you please introduce yourself and explain your role to him?

*Interpreter: The interpreter is expected to explain her/his role here to both parties. The next turn will need to be adapted according to what the interpreter says.*

Suspect: Oh, that’s good, thanks for coming. I was worried I wouldn’t be able to communicate with the police, and I know that can be quite dangerous

POLICE: Ok, thank you. My name is Detective Inspector Costa and I’m attached to the Joint Counter Terrorism Team. We also have a Mandarin interpreter with us, who will interpret everything from and into English. Are you able to understand the interpreter?

SUSPECT: Yes, of course, I understand him/her perfectly.

POLICE: First of all, I have to tell you that we need to ask you certain questions, so I must caution you that you don’t have to say or do anything, but everything anything that you do say or do will be recorded and may be used in evidence. Do you understand?

SUSPECT: What do you mean? are you already sending me to court? but why? what have I done? I want answers. I haven’t done anything wrong, so I don’t understand why I’m being placed in such an uncomfortable situation. I’m very tired after such a long flight and I want to go home.

POLICE: Before we continue, you say that you are very tired. Are you ok to continue with the interview now? Or should we give you some time to rest?

SUSPECT: No, no, I want to leave as soon as possible, so please let’s get on with it so I can leave

POLICE: Ok, good., But, but first it is very important for me to know that you understand the official caution I have given you. Can you please explain it back to me in your own words?

SUSPECT: Well, yes, that I don’t have to say anything I don’t want, and that what I say can be used in court, is that it?

POLICE: Yes, that’s right, Now I will tell you what your rights are: You have the right to legal representation. Would you like to contact a lawyer?

SUSPECT: Well, uh, no, I don’t have a lawyer.

POLICE: Ok. You also have the right to speak with a consular official or a support person, a relative or friend. Would you like to contact anyone before we start?

SUSPECT: No, please get on with it, I’m getting more nervous by the minute

POLICE: Ok, no need to get nervous. If at any time during the interview you decide that you want to contact a lawyer, friend, relative or consular official, please let me know and we will suspend the interview and help you contact one. Ok?

SUSPECT: Ok, very kind of you.

POLICE: Also, there is a video camera over there recording everything. At the conclusion of the interview you will be provided with acopy of the entire interview.

SUSPECT: Ah, that’s good, in case I need it.

POLICE: If at any time you don’t understand a question that’s been asked, please let us know, ok? Also, if you ever need a break, water or food, let me know. And are you well enough to continue the interview?

SUSPECT: Ah, Ok, I’ll let you know if I don’t understand anything, and don’t worry, I’m ok, let’s get on with it.

Police: Ok, thank you. Now I can tell you that we’re investigating your involvement in a transnational criminal syndicate, which is believed to be financing a terrorist organisation.

SUSPECT: What? That’s crazy, I know him, but I have nothing to do with anything criminal!

POLICE: Now, can I ask you to please state your full name, address and date of birth?

SUSPECT: Ok, my name is (Add Chinese name), but people call me Ronny, and now I live at 3/15 Flora St, Liverpool I was born on 3/2/1969. (24).

POLICE: Right, and what is your occupation?

SUSPECT: Uh, I’m a brikkie’s labourer, I work in the construction industry, helping out wherever is needed.

Police: Ah, okay, Ronny. Tell me about your work at the moment then. How long have you been working there? What exactly do you do? Who do you report to?

SUSPECT: Uh, well, a friend of mine used to work there and he knew I was looking for a job so he told his foreman and he said ok, ask him to come, we need a labourer, so I did and there I am now, uh, I think I’ve been working there for about 3 years? Uh, I don’t know the full name of the foreman, we call him Jim.

POLICE: Ok, that’s great, thanks. Isn’t it good that your friend recommended you and you got a job? Do you enjoy working there?

SUSPECT: Yeah, it’s a job…

POLICE: Ok, good. And, and could you please tell us your nationality?

SUSPECT: I’m Chinese, I was born in mainland China.

POLICE: Right, and do you travel back to often?

SUSPECT: Uhm, well, yes, quite often, uh, but I really don’t know what you mean by ‘often’, I mean, I go maybe once a year to see my family, my mum in particular.

POLICE: That’s good that you go and see your mum often. I should go and visit my mum more myself! !

And do you travel to other countries apart from China? You travel to other Asian countries as well quite often, don’t you? (42)?

SUSPECT: Uh, well, yes. Why? is that illegal?

POLICE: No, that’s not illegal. We just need to know what other countries you travel to and the reasons why.

SUSPECT: Well, why? don’t I have the right to visit other countries! ?

Police: Mr (add surname), I ask the question. What other Asian countries have you travelled to in the past twelve months?

Suspect: Well, yes, in the past 12 months I’ve travelled to other countries, I’ve been to Thailand, Laos, Vietnam, Taiwan, Indonesia, lots of places, I love travelling. Have you ever been to Asia yourself?

Police: No, actually, I’d love to travel and visit those coutnries! Now, I’m curious. How, how did you get the money to travel so much on a brickies labourer’s income?

SUSPECT: Well, uh, I’ve been saving the money. I live on my own and don’t spend much money. Actually, that’s all I spend my money on, what else is there for a single man to do?

***SPLIT HERE – (approx. 1000 words) – Police stops the interview***

POLICE: You see, Ronny, your story is a bit unlikely, because you came back to Australia after visiting each of these countries, and you only stayed at each of them for less than a week. And then you go back again after a week or so in Australia.

SUSPECT: Yes, that’s because I had to come back to Australia to work. I couldn’t take more than a week off at a time, and I need to earn more money to cover all the expenses.

*(To the interpreter)*: I don’t like the way he’s asking these questions, he’s trying to insinuate that I’ve done something wrong, and that’s not true. Don't tell him this, but I need to talk to you because you speak my language and you can help me.

POLICE: *(The police will respond differently depending on what the interpreter did with the previous segment)*

*Option 1 –* if the interpreter interpreted everything faithfully:

Mr (add surname), I warn you that you can’t engage the interpreter in conversation. The interpreter is impartial and is here to interpret everything that is said by you and by me.

*Option 2 –* if the interpreter doesn’t interpret everything and it is obvious:

Sorry interpreter, can you please make sure you interpret everything that is said?

POLICE: Now, Ronny, do you know a Mr Ahmad Ayoub who resides in Australia?

POLICESuspect: Yes, of course, he works with me on the building site, he’s a labourer like me.

POLICE: And you’re good mates, aren’t you?

SUSPECT: Sí, más o menos. aunque no nos entendemos muy bien porque ninguno habla inglés, él habla árabe y yo español, solo que trabajamos juntos en las obras, pero la verdad es que no tenemos mucho en común, a veces almorzábamos juntos. (41)

SUSPECT: yeah, pretty much. Although we don’t understand each other very well, because neither of us speaks English very well. He speaks Arabic and I speak Chinese, we just work on the same construction sites, but I wouldn’t say we have much in common with each other. Sometimes we have lunch together.

POLICE: And did you ever talk about politics or religion? Did he ever tell you what his plans were for the future?

SUSPECT: Ah, no, not really. He used to say he didn’t like Australia much and that he wanted to go back home to help his people, so he was working hard to save money to help the poor people in his country, but I don’t really know what he meant by that. I think he was Muslim, but I don't really know. I didn’t care what religion he was. I don't want any trouble, you know?

Police: Ronny, we don’t want you to get into any trouble either. Now, did you know that he went to Syria to fight in the jihadist insurgency? (26)?

SUSPECT: Uh, no, I can’t believe that, are you sure? I didn’t think he was that type of person. Are you saying he’s a terrorist? Gee that’s scary! You need to believe me, I didn't know anything! Now I understand why he hasn’t turned up to work for a while.

Police: But, Ronny, I think you knew him better than what you want to admit. Didn’t he keep in touch with you? Didn’t he send you emails from there?

SUSPECT: Uh, well, I didn’t know where he was sending them from, and everyone gets unwanted emails from all over the place. I often get emails from Nigeria and Russia asking me for money, I’m sure you do too, that doesn’t mean I’m connected to them.

Police: Ok, Ronny, yes, we all do, don’t we? Now, Now, Ronny, I believe you are a Facebook friend of Ahmad? Aren’t you?

SUSPECT: Uh, well, yes, I think so, I don't know really. I can’t keep track of all the Facebook friends, most of them I don’t know, they’re friends of friends. I was thinking of getting rid of my Facebook account. It’s nothing but a nuisance, and now this confirms it!

POLICE: And, have you seen his latest posts on his wall where he’s posted photos of himself in jihadist uniform fighting in Syria?

SUSPECT: Uh, no, frankly I haven’t been on Facebook for a while

POLICE: Ok. Now, do you know a Mr Huang who resides in China?

SUSPECT: Yes, I know him

POLICE: And, can you tell me about how you know him?

SUSPECT: Well, uh, he was introduced to me by another friend who travelled with me to China last time. Why? Is he in Syria as well?

POLICE: We have reason to believe that he has contacts in Syria through you. Have you introduced Huang to Ahmad?

SUSPECT: No….no…Why would I? They don’t even speak the same language or belong to the same religion

POLICE: That’s why you are useful to them, Ronny, because you can sort of translate for them.

SUSPECT: No way! How can I translate when I can hardly speak English!

POLICE: You see Ronny, we have reason to believe that you may be part of a money laundering cell in Australia

SUSPECT: No way! I don’t even know what that means!

*– To the interpreter –* Can you please tell him I’m innocent? I can’t stand this for much longer! I want to go home now. They can’t keep me here like this.

POLICE: Now, Ronny, please calm down. I can tell you that the Chinese drug police has raided Huang’s house and confiscated $10,000 worth of mythelamphetamine. Did you know about that?

SUSPECT: No… how would I know that? I told you I don’t know that bloke well

Police: Then how do you explain that among his bank transactions there was one made to your bank account for $100,000? And futhermore, that you then made a transfer to Ahmad’s account for $90,000?

SUSPECT: Uh, look, I’m not feeling well now and I think I can’t answer any more questions, I think I need to call a solicitor, but I don’t know who to call

POLICE: Ok Ronny, we’ll suspend the interview now to let you find a solicitor. Here’s a list of solicitors available to you for the purpose of providing advice. Please choose one and we will make arrangements to put you in touch with the solicitor of your choosing.

SUSPECT: Look, in that case, let’s just finish the interview, because I want to finish now. I don’t know who to call.

POLICE: Ok, then. Mr Huang, I am now formally charging you with being knowingly involved in money laundering with the purpose of aiding and abetting terrorist activity. You will be summoned to go to court to answer the charge where you can plead guilty or not guilty.

SUSPECT: No, no, no, I can’t believe this! What have I got myself into?!

POLICE: Now, this concludes the interview. Have you given your answers of your own free will and choice?

SUSPECT: Yes, yes.

POLICE: Has anyone made any threat, promise or inducement for you to give your answers?

SUSPECT: No, no, please let me go now.

POLICE: Ok, Ronny, that concludes the interview. Thank you Madam/Mr Interpreter for your excellent work.

SUSPECT: Ok. Thank you.

(End of script)
