# Supplementary material for: An Eye-Movement Analysis of Overt Visual Attention During Consecutive and Simultaneous Interpreting Modes in a Remotely Interpreted Investigative Interview
Source: Front Psychol. 2022 Mar 25;13:764460. doi: 10.3389/fpsyg.2022.764460 (PMC8992695; doi:10.3389/fpsyg.2022.764460)
Supplement: Supplementary file 2 [file Data_Sheet_2.docx]

Police interview

POLICE: (To interpreter) Hello Mr/Madam Interpreter. I’m glad you were able to come on time. We’ll be interviewing this man who we understand speaks only Arabic and can’t speak English, so we need you to interpret for us. Can you please introduce yourself and explain your role to him?

*Interpreter: The interpreter is expected to explain her/his role here to both parties. The next turn will need to be adapted according to what the interpreter says.*

SUSPECT: Oh, that’s good, thanks for coming. I was worried I wouldn’t be able to communicate with the police, and I know that can be quite dangerous

آه، هذا جيّد، شكرا" لمجيئك. كنت قلقا" لأنني لن يمكنني التواصل مع الشرطة، وانا اعلم انا هذا قد يكون خطير جدا".

POLICE: Ok, thank you. My name is Detective Inspector Costa and I’m attached to the Joint Counter Terrorism Team. We also have an Arabic interpreter with us, who will interpret everything from and into English. Are you able to understand the interpreter?

SUSPECT: Yes, of course, I understand him/her perfectly.

نعم، بالطبع، أفهم عليه بشكل ممتاز.

POLICE: First of all, I have to tell you that we need to ask you certain questions, so I must caution you that you don’t have to say or do anything, but everything that you do say or do will be recorded and may be used in evidence. Do you understand?

SUSPECT: What do you mean? are you already sending me to court? but why? what have I done? I want answers. I haven’t done anything wrong, so I don’t understand why I’m being placed in such an uncomfortable situation. I’m very tired after such a long flight and I want to go home.

المشتبه به: ماذا تقصد؟هل سترسلني من الآن الى المحكمة؟ ولكن لماذا؟ماذا فعلت؟ أريد أجوبة. لم أفعل شيئا" خطأ، لذا لا أعلم لماذا تمّ وضعي في موقف غير مريح كهذا. أشعر بتعب شديد بعد سفرة طويلة كهذه واريد أن أذهب الى المنزل.

POLICE: Before we continue, you say that you are very tired. Are you ok to continue with the interview now? Or should we give you some time to rest?

SUSPECT: No, no, I want to leave as soon as possible, so please let’s get on with it so I can leave

كلا، كلا، اريد ان اغادر بأقرب وقت ممكن، لذا دعونا نتابع لكي أغادر.

POLICE: Ok, good. But but first it is very important for me to know that you understand the official caution I have given you. Can you please explain it back to me in your own words?

SUSPECT: Well, yes, that I don’t have to say anything I don’t want, and that what I say can be used in court, is that it?

المشتبه به: حسنا، نعم، أنني لست مجبرا" على قول أي شيء لا أرغب في قوله، وأنّ ما أقوله يمكن أن يستخدم في المحكمة، أليس كذلك؟

POLICE: Yes, that’s right, Now I will tell you what your rights are: You have the right to legal representation. Would you like to contact a lawyer?

SUSPECT: Well, uh, no, I don’t have a lawyer.

المشتبه به: حسنا"، اه، كلا، ليس لدي محام.

POLICE: Ok. You also have the right to speak with a consular official or a support person, a relative or friend. Would you like to contact anyone before we start?

SUSPECT: No, please get on with it, I’m getting more nervous by the minute

المشتبه به: لا، من فضلك أكمل، مع كل دقيقة تمر أشعر بالتوتر أكثر فأكثر.

POLICE: Ok, no need to get nervous. If at any time during the interview you decide that you want to contact a lawyer, friend, relative or consular official, please let me know and we will suspend the interview and help you contact one. Ok?

SUSPECT: Ok, very kind of you.

المشتبه به: حسنا"، هذا لطف كبير منك.

POLICE: Also, there is a video camera over there recording everything. At the conclusion of the interview you will be provided with a copy of the entire interview.

SUSPECT: Ah, that’s good, in case I need it.

المشتبه به: ايه، هذا جيد، في حال كنت بحاجة لها.

POLICE: If at any time you don’t understand a question that’s been asked, please let us know, ok? Also, if you ever need a break, water or food, let me know. And are you well enough to continue the interview?

SUSPECT: Ah, Ok, I’ll let you know if I don’t understand anything, and don’t worry, I’m ok, let’s get on with it.

المشتبه به: آه، حسنا"، سوف أعلمك إذا لم أفهم شيئا" ما، ولا تقلق، أنا بحالة جيّدة، دعنا نكمل.

POLICE: Ok, thank you. Now I can tell you that we’re investigating your involvement in a transnational criminal syndicate, which is believed to be financing a terrorist organisation.

SUSPECT: What? That’s crazy! I have nothing to do with anything criminal!

مشتبه به: ماذا؟ هذا جنون، أعرفه ولكن ليس لدي أي علاقة بأي شيء جرمي!

POLICE: Now, can I ask you to please state your full name, address and date of birth?

SUSPECT: Ok, my name is Samir Abboud, but people call me Ronny, and now I live at 3/15 Flora St, Liverpool I was born on 3/2/1969.

المشتبه به: حسنا"، اسمي سمير عبود ولكن الناس ينادوني ب"روني"، وأنا أسكن الآن على العنوان 3/15 شارع فلورا، ليفربول وولدت في 3/2/1969.

POLICE: Right, and what is your occupation?

الشرطي: حسنا"، وما هي وظيفتك؟

SUSPECT: Uh, I’m a brickie’s labourer, I work in the construction industry, helping out wherever is needed.

مشتبه به: آه، انا عامل في مجال بناء الحجر، اعمل في مجال البناء، اساعد اينما كان هناك حاجة للمساعدة.

POLICE: Ah, okay, Ronny. Tell me about your work at the moment then. How long have you been working there? What exactly do you do? Who do you report to?

SUSPECT: Uh, well, a friend of mine used to work there and he knew I was looking for a job so he told his foreman and he said ok, ask him to come, we need a labourer, so I did and there I am now, uh, I think I’ve been working there for about 3 years? Uh, I don’t know the full name of the foreman, we call him Jim.

المشتبه به: ايه، حسنا"، أحد اصدقائي كان يعمل هناك وكان يعلم انني ابحث عن عمل فقال للمسؤول عنه وقال نعم، اطلب منه المجيء، نحن بحاجة الى عامل، فقمت بذلك، وها انا اليوم، ايه، اعتقد انني أعمل هناك لحوالي ثلاث سنوات. ايه، لا أعلم اسم المسؤول الكامل ولكننا ندعوه جيم.

POLICE: Ok, that’s great, thanks. Isn’t it good that your friend recommended you and you got a job? Do you enjoy working there?

SUSPECT: Yeah, it’s a job…

المشتبه به: نعم، إنّه عمل...

POLICE: Ok, good. And, and could you please tell us your nationality?

الشرطي: وهل يمكنك أن تقول لنا ما هي جنسيّتك؟

SUSPECT: I’m Lebanese, I was born in Lebanon.

المشتبه به: أنا لبناني، ولدت في لبنان.

POLICE: Right, and do you travel back to Lebanon often?

الشرطي: حسنا"، هل تسافر الى لبنان كثيرا"؟

SUSPECT: Uhm, well, yes, quite often, uh, I really don’t know what you mean by ‘often’, I mean, I go maybe once a year to see my family, my mum in particular.

المشتبه به: أوم، حسنا، نعم، كثيرا" جدا"، آه، أنا حقاً لا أعرف ماذا تقصد 'بكثيرا'، يعني، اذهب ربما مرة في السنة لرؤية عائلتي، أمي بالتحديد.

POLICE: That’s good that you go and see your mum often. I should go and visit my mum more myself!

And do you travel to other countries apart from Lebanon? You travel to other Middle Eastern countries as well quite often, don’t you? (42)?

الشرطي: وهل تسافر إلى بلدان أخرى غير لبنان؟ تسافر إلى دول شرق أوسطيّة أخرى أيضا"، اليس كذلك؟

SUSPECT: Uh, well, yes. Why? is that illegal?

المشتبه به: حسنا" آه، نعم. لماذا؟ هل هذا مخالف للقانون؟

POLICE: No, that’s not illegal. We just need to know what other countries you travel to and the reasons why.

الشرطي: كلا هذا ليس مخالفا" للقانون، علينا فقط أن نعلم اذا كنت تسافر الى بلدان أخرى وما هو سبب ذلك؟

SUSPECT: Well, why? don’t I have the bloody right to visit those countries?

المشتبه به: حسنا"، لماذا؟ أليس لدي الحق النحس بزيارة تلك البلدان؟

Mr Abboud, I ask the question. What other Middle Eastern countries have you travelled to in the past twelve months?

SUSPECT: Well, yes, in the past 12 months I’ve travelled to other countries, I’ve been to Dubai, Egypt, Morocco, Tunisia, Iraq, lots of places, I love travelling. Have you ever been to the Middle East yourself?

المشتبه به: حسنا"، نعم، سافرت الى بلدان أخرى في خلال الإثني عشر شهر الماضي، سافرت الى دبي ومصروالمغرب وتونس والعراق وأماكن عديدة، أحب السفر. هل سبق وسافرت الى الشرق الأوسط انت؟

POLICE: No, actually, I’d love to travel and visit those countries ! Now, I’m curious. How did you get the money to travel so much on a brickie’s labourer’s income?

SUSPECT: Well, uh, I’ve been saving the money. I live on my own and don’t spend much money. Actually, that all I spend my money on, what else is there to do for a single man?

المشتبه به: حسنا، آه، لقد كنت أصمّد المال. أعيش بمفردي ولا انفق الكثير من المال. في الواقع، هذا كل ما أنفق اموالي عليه، ماذا يمكنني القيام به غير ذلك كرجل أعزب؟

***SPLIT HERE – (approx. 1000 words) – Police 2 stops the interview***

POLICE: You see, Ronny, your story is a bit unlikely, because you came back to Australia after visiting each of these countries, and you only stayed at each of them for less than a week. And then you go back again after a week or so in Australia.

الشرطي: هل ترى روني، قصتك من المرجّح أنها غير صحيحة، لأنّك عدت الى أستراليا بعد زيارة كل بلد من تلك البلدان، وبقيت في كلّ منها أقل من أسبوع.

SUSPECT: Yes, that’s because I had to come back to Australia to work. I couldn’t take more than a week off at a time, and I need to earn more money to cover all the expenses.

المشتبه به: نعم، هذا لأنه كان علي العودة الى أستراليا للعمل. لم يكن بإمكاني أن آخذ أكثر من أسبوع إجازة في وقت واحد، وانا بحاجة لكسب المزيد من المال لتغطية جميع النفقات.

*(To the interpreter)*: I don’t like the way he’s asking these questions, he’s trying to insinuate that I’ve done something wrong, and that’s not true. Don't tell him this, but I need to talk to you because you speak my language and you can help me.

(للمترجم): لا تعجبني الطريقة التي يطرح فيها هذه الأسئلة، إنّه يحاول أن يلمّح بأنني قد فعلت شيئا خطأ، وهذا ليس صحيحاً. لا تقول له هذا، ولكن أنا بحاجة إلى أن أتحدث إليك لأنّك تتكلم لغتي ويمكنك مساعدتي.

POLICE: *(The police will respond differently depending on what the interpreter did with the previous segment)*

*Option 1 –* if the interpreter interpreted everything faithfully: Mr Abboud, I warn you that you can’t engage the interpreter in conversation. The interpreter is impartial and is here to interpret everything that is said by you and by me.

الخيار 1_أستاذ(أضف إسم العائلة)، أحذّرك أنّك لا يمكنك أن تشرك المترجم بالحديث. المترجم محايد وهو موجود هنا لكي يترجم كل شيء تقوله وكل شيء أنا أقوله.

*Option 2 –* if the interpreter doesn’t interpret everything and it is obvious to the police: Sorry interpreter, can you please make sure you interpret everything that is said?

الخيار 2_إذا لم يترجم المترجم كل شيء وكان الأمر واضح بالنسبة للشرطي: عفوا" أيها المترجم، هل يمكنك من فضلك أن تتأكّد من أن تترجم كل شيء يقال.

POLICE: Now, Ronny, do you know a Mr Ahmad Ayoub who resides in Australia?

الشرطي: طيّب، روني، هل تعرف شخص باسم السيد أحمد أيوب وهو يقيم في أستراليا؟

SUSPECT: Yes, of course, he works with me on the building site, he’s a bricky like me.

المشتبه به: نعم، بالطبع، إنه يعمل معي في مقرّ البناء، وهو بنّاء حجر مثلي.

POLICE: And you’re good mates, aren’t you?

الشرطي: وأنتم أعزالأصحاب، أليس كذلك؟

SUSPECT: yeah, pretty much. Although we don’t understand each other very well, because he speaks a different dialect from mine, he’s from Syria, we just work on the same construction sites, but I wouldn’t say we have much in common with each other. Sometimes we have lunch together.

المشتبه به: نعم، شيء من هذا القبيل. على الرغم من أننا لا نفهم على بعضنا البعض كثيرا"، لأنه يتحدث بلهجة مختلفة عن لهجتي، هو من سوريا، لا يجمعني به إلّا مجرّد أننا نعمل في مقر البناء نفسه ولكن لا أعتبر أن لدينا الكثير من القواسم المشتركة مع بعضنا البعض. في بعض الأحيان نتناول طعام الغداء معا.

POLICE: And did you ever talk about politics or religion? Did he ever tell you what his plans for the future were?

الشرطي: وهل سبق وتكلّمت معه حول السياسة أو الدين؟ هل سبق وقال لك ما هي مخطّطاته المستقبليّة؟

SUSPECT: Ah, no, not really. He used to say he didn’t like Australia much and that he wanted to go back home to help his people, so he was working hard to save money to help the poor people in his country, but I don’t really know what he meant by that. I know he’s Muslim, but that doesn’t mean he’s a terrorist! I have heaps of Muslim friends and they’re excellent people who wouldn’t harm anyone. I didn’t care what religion he was. I don't want any trouble, you know?

المشتبه فيه: حسنا"، لا، ليس كثيرا". كان يقول أنّه لا يحب أستراليا كثيرا وأنّه يريد العودة الى وطنه الأم لمساعدة شعبه، فكان يعمل جاهدا لتصميد المال لمساعدة الفقراء في بلده، ولكن أنا لست على يقين ما الذي كان يقصده بذلك. أنا أعرف أنّه مسلم، ولكن هذا لا يعني أنّه إرهابي! لدي عدد كبير من الأصدقاء المسلمين وهم ممتازون ولا يأذون أحدا". لم يهمّني ما هي ديانته. أنا لا أريد أي مشاكل، عرفت؟

POLICE: Ronny, we don’t want you to get into any trouble either. Now, did you know that he went to Syria to fight in the jihadist insurgency?

الشرطي: روني، نحن أيضا" لا نريدك أن تتورّط في أي مشاكل. والآن، هل تعلم أنّه ذهب إلى سوريا للمحاربة في صفوف الجهاديين المتمرّدين؟

SUSPECT: Uh, no, I can’t believe that, are you sure? I didn’t think he was that type of person. Are you saying he’s a terrorist? Gee that’s scary! You need to believe me, I didn't know anything! Now I understand why he hasn’t turned up to work for a while.

المشتبه به: آه، لا، لا أصدق ذلك، هل أنت متأكد؟ لم أكن أعتقد أنه من ذلك النوع من الأشخاص. هل أنت تقول أنه إرهابي؟ يا الهي هذا أمر مخيف! يجب ان تصدّقني، لم أكن أعرف أي شيء! والآن أنا أفهم سبب تغيّبه عن العمل لفترة طويلة.

POLICE: Yes, that’s right. But, Ronny, I think you knew him better than what you want to admit. Didn’t he keep in touch with you? Didn’t he send you emails from there?

الشرطي: نعم، هذا صحيح. ولكن، روني، أعتقد أنك تعرفه أكثر مما تريد أن تقر. ألم يبقى على اتصال بك؟ ألم يرسل لك رسائل عبر البريد الإلكتروني من هناك؟

SUSPECT: Uh, well, I didn’t know where he was sending them from, and everyone gets unwanted emails from all over the place. I often get emails from Nigeria and Russia asking me for money, I’m sure you do too, that doesn’t mean I’m connected to them.

المشتبه به: آه، حسنا، كيف تعرف أنّه أرسل لي رسائل عبر البريد الإلكتروني؟ وعدى عن ذلك، لم أكن أعرف من أين كان يرسلها لي، وكل شخص يحصل على رسائل عبر البريد الإلكتروني غير مرغوب فيها ومن كل المكان. وكثيراً ما احصل على رسائل عبر البريد الإلكتروني من نيجيريا وروسيا يطلبون فيها مني المال، وأنا متأكد من أنّه يصلك نفس الرسائل، هذا لا يعني أنّني على علاقة بهم.

POLICE: Yes, we all get those emails, don’t we? Now, Ronny, I believe you are a Facebook friend of Ahmad’s? aren’t you?

الشرطي: حسنا" روني، أنا أعتقد أنّك صديق أحمد على ال"فايسبوك"؟ أليس كذلك؟

SUSPECT: Uh, well, yes, I think so, I don't know really. I can’t keep track of all the Facebook friends, most of them I don’t know, they’re friends of friends. I was thinking of getting rid of that bloody Facebook account! It’s nothing but a nuisance, and now this confirms it!

المشتبه به: ايه، حسنا"، نعم، أعتقد ذلك، لست متأكّدا". لا أستطيع أن أتعقّب جميع أصدقائي على فيسبوك، معظمهم لا أعرفهم، هم أصدقاء أصدقائي. كنت أفكّر أن أتخلّص من حساب فايسبوك النحس. فهو ليس سوى مصدر إزعاج، وهذا خير دليل على ذلك!

POLICE: And, have you seen his latest posts on his wall where he’s uploaded photos of himself in jihadist uniform fighting in Syria?

الشرطي: وهل رأيت ما نشره مؤخرا" على صفحته حيث نشرصورا" له في زي القتال الّذي يرتدونه الجهاديين في سوريا؟

SUSPECT: Uh, no, frankly I haven’t been on Facebook for a while

المشتبه به: أه، كلا، بصراحة لم أدخل على فيسبوك لفترة طويلة.

POLICE: Ok. Now, do you know a Mr Xin who resides in Dubai?

الشرطي: حسنا". والآن، هل تعرف شخص بإسم السيّد "شن" وهو يسكن في دبي؟

SUSPECT: Yes, I know him. He’s a Chinese businessman who lives in Dubai.

المشتبه به: نعم، أعرفه، هو رجل أعمال صيني يسكن الآن في دبي.

POLICE: And, how do you know him?

الشرطي: وكيف تعرفت عليه؟

SUSPECT: Well, uh, he was introduced to me by another friend who travelled with me to Dubai last time. Why? Is he in Syria as well?

المشتبه به: حسنا"، ايه، عرّفني عليه صديق آخر كان قد سافر معي الى دبي المرّة السابقة. لماذا؟ هل هو أيضا" في سوريا؟

POLICE: We have reason to believe that he has contacts in Syria through you. Have you introduced Mr Xin to Ahmad?

الشرطي: كلا، هو لا يزال في دبي، ولكن لدينا ما يجعلنا نعتقد أنّه لديه معارف بسوريا من خلالك. هل عرّفت الأستاذ "شن" على أحمد.

SUSPECT: No….no…Why would I? They don’t even speak the same language or belong to the same religion.

المشتبه به: كلا...كلا...لماذا أقدم على ذلك؟ حتّى أنّهم لا يتكلّمون اللغة نفسها ولا ينتمون الى الديانة نفسها.

POLICE: That’s why you are useful to them, because you can sort of translate for them.

الشرطي: لذا انت مفيدا" لهم، لأنّك يمكنك نوعا" ما أن تترجم لهم.

SUSPECT: No way! How can I translate when I can hardly speak English and I don’t speak Chinese either!

المشتبه به: غير معقول! كيف يمكنني الترجمة وأنا بالكاد أستطيع تكلّم الإنجليزية ولا أتكلم اللغة الصينية.

POLICE: You see Ronny, we have reason to believe that you may be part of a money laundering cell in Australia.

الشرطة: هل تعلم روني، لدينا ما يجعلنا نعتقد بأنك قد تكون جزءا" من خلية غسل أموال في أستراليا.

SUSPECT: No way! I don’t even know what that means!

المشتبه به: غير ممكن! حتى أنني لا أعلم ما معنى ذلك؟

*– To the interpreter – Can you please tell him I’m innocent? I can’t stand this for much longer! I want to go home now. They can’t keep me here like this.*

*-للمترجم- هل يمكنك أن تقول له أنني بريء؟ لا يمكنني أن أتحمّل هذا لفترة أطول! أريد أن أذهب الى المنزل الآن. لا يمكنهم أن يبقوني هنا هكذا.*

POLICE: Now, Ronny, please calm down. I can tell you that the Dubai drug police has raided Mr Xin’s house and confiscated $10,000 worth of methylamphetamine. Did you know about that?

الشرطي: الآن، روني، من فضلك إهدأ. يمكنني أن أقول لك أنّ شرطة مكافحة المخدرات في دبي داهمت منزل السيّد "شين" وصادرت ميثامفيتامين بقيمة 10000 دولار. هل أنت على علم بذلك؟

SUSPECT: No… how would I know that? I told you I don’t know that bloke well

المشتبه به: كلا.. كيف لي أن أعلم ذلك؟ قلت لك أنني لا أعرف هذا الرجل جيّدا".

POLICE: Then how do you explain that among his bank transactions there was one made to your bank account for $100,000? And furthermore, that you then made a transfer to Ahmad’s account for $90,000?

الشرطي: إذا كيف تفسّر أنّه من بين معاملاته المصرفية كان هناك تحويل الى حسابك المصرفي بمبلغ 100000 دولار؟ وبالإضافة الى ذلك، أنّك حوّلت بعدها مبلغ 90000 دولار الى حساب أحمد؟

SUSPECT: Uh, look, I’m not feeling well now and I think I can’t answer any more questions, I think I need to call a solicitor, but I don’t know who to call

*المشتبه به: ايه، أنظر، أشعر انني لست على ما يرام وأظن أنّه لا يمكنني أن أجيب على أي أسئلة إضافيّة، أعتقد أنّه علي أن أتّصل بمحام، ولكن لا أعلم بمن أتّصل-الى المترجم-هل يمكنك من فضلك أن تساعدني للعثور على محام يتكلّم اللغة العربيّة؟ يجب أن يكون بإمكانك أن ترشدني الى محام جيّد، من فضلك أنا بحاجة لمساعدتك.*

POLICE: Ok Ronny, we’ll suspend the interview now to let you find a solicitor. Here’s a list of solicitors available to you for the purpose of providing advice. Please choose one and we will make arrangements to put you in touch with the solicitor of your choosing.

SUSPECT: Look, in that case, let’s just finish the interview, because I want to go now. I don’t know who to call.

المشتبه به: أنظر، في هذه الحالة، دعنا ننهي الإستجواب، لأنني أريد ان أغادر الآن. لا أعلم بمن سأتّصل.

POLICE: Ok, then. Mr Abboud, I am now formally charging you with being knowingly involved in money laundering with the purpose of aiding and abetting terrorist activity. You will be summoned to go to court to answer the charge where you can plead guilty or not guilty.

SUSPECT: No, no, no, I can’t believe this! What have I got myself into?!

المشتبه به: لا، لا، لا، لا أستطيع أن أصدّق هذا! بماذا ورّطت نفسي؟!

POLICE: Have you given your answers of your own free will and choice?

الشرطي: هل أدليت بأجوبتك بمحض إرادتك وإختيارك؟

SUSPECT: Yes, yes.

المشتبه به: نعم، نعم.

POLICE: Has anyone made any threat, promise or inducement for you to give your answers?

الشرطي: هل قام أي شخص بتهديدك أو تقديم وعود لك أو تشجيعك لكي تدلي بأجوبتك؟

SUSPECT: No, no, please let me go now.

المشتبه به: كلا، كلا، من فضلك دعني أغادر الآن.

POLICE: Ok, Ronny, that concludes the interview. Thank you Madam/Mr Interpreter for your excellent work.

SUSPECT: Ok. Thank you.

(End of script )
