# Supplementary material for: An Eye-Movement Analysis of Overt Visual Attention During Consecutive and Simultaneous Interpreting Modes in a Remotely Interpreted Investigative Interview
Source: Front Psychol. 2022 Mar 25;13:764460. doi: 10.3389/fpsyg.2022.764460 (PMC8992695; doi:10.3389/fpsyg.2022.764460)
Supplement: Supplementary file 3 [file Data_Sheet_3.docx]

Police interview

POLICE: (To interpreter) Hello Mr/Madam Interpreter. I’m glad you were able to come on time. We’ll be interviewing this man who we understand speaks only Mandarin and can’t speak English, so we need you to interpret for us. Can you please introduce yourself and explain your role to him?

*Interpreter: The interpreter is expected to explain her/his role here to both parties. The next turn will need to be adapted according to what the interpreter says.*

Suspect: Oh, that’s good, thanks for coming. I was worried I wouldn’t be able to communicate with the police, and I know that can be quite dangerous（那太好了，谢谢你。刚才我还在担心跟警察说不清楚，那样的话太危险了。）

POLICE: Ok, thank you. My name is Detective Inspector Costa and I’m attached to the Joint Counter Terrorism Team. We also have a Mandarin interpreter with us, who will interpret everything from and into English. Are you able to understand the interpreter?

SUSPECT: Yes, of course, I understand him/her perfectly. (当然可以，完全能听明白。)

POLICE: First of all, I have to tell you that we need to ask you certain questions, so I must caution you that you don’t have to say or do anything, but everything anything that you do say or do will be recorded and may be used in evidence. Do you understand?

SUSPECT: What do you mean? are you already sending me to court? but why? what have I done? I want answers. I haven’t done anything wrong, so I don’t understand why I’m being placed in such an uncomfortable situation. I’m very tired after such a long flight and I want to go home. （你什么意思？现在就要把我带到法院吗？为什么？我做了什么？你得说清楚。我又没干什么坏事，我不明白你们为什么要让我这么难受。我坐长途飞机过来已经很累了，我想回家。）

POLICE: Before we continue, you say that you are very tired. Are you ok to continue with the interview now? Or should we give you some time to rest?

SUSPECT: No, no, I want to leave as soon as possible, so please let’s get on with it so I can leave （不用不用，我只想尽快离开这里，我们开始吧，尽量早点做完。）

POLICE: Ok, good., But, but first it is very important for me to know that you understand the official caution I have given you. Can you please explain it back to me in your own words?

SUSPECT: Well, yes, that I don’t have to say anything I don’t want, and that what I say can be used in court, is that it? （那行吧，就是说如果我不愿意的话，可以不用说，对吗？）

POLICE: Yes, that’s right. Now I will tell you what your rights are: You have the right to legal representation. Would you like to contact a lawyer?

SUSPECT: Well, uh, no, I don’t have a lawyer.（这个嘛，嗯，没有，我没有找律师。）

POLICE: Ok. You also have the right to speak with a consular official or a support person, a relative or friend. Would you like to contact anyone before we start?

SUSPECT: No, please get on with it, I’m getting more nervous by the minute（不用，快开始吧，搞得我越来越紧张。）

POLICE: Ok, no need to get nervous. If at any time during the interview you decide that you want to contact a lawyer, friend, relative or consular official, please let me know and we will suspend the interview and help you contact one. Ok?

SUSPECT: Ok, very kind of you.（好的，很谢谢你。）

POLICE: Also, there is a video camera over there recording everything. At the conclusion of the interview you will be provided with a copy of the entire interview.

SUSPECT: Ah, that’s good, in case I need it. （哦那好，万一我需要呢）

POLICE: If at any time you don’t understand a question that’s been asked, please let us know, ok? Also, if you ever need a break, water or food, let me know. And are you well enough to continue the interview?

SUSPECT: Ah, Ok, I’ll let you know if I don’t understand anything, and don’t worry, I’m ok, let’s get on with it.（嗯好的，如果有不明白的地方我就跟你说，别担心，我没事，继续吧。）

Police: Ok, thank you. Now I can tell you that we’re investigating your involvement in a transnational criminal syndicate, which is believed to be financing a terrorist organisation.

SUSPECT: What? That’s crazy, I have nothing to do with anything criminal!（什么？太扯了，违法的事绝对跟我无关！）

POLICE: Now, can I ask you to please state your full name, address and date of birth?

SUSPECT: Ok, my name is Rong Chen, but people call me Ronny, and now I live at 3/15 Flora St, Liverpool I was born on 3/2/1969. (24). （好吧。我叫陈荣，大家都叫我Ronny，我家地址是3/15 Flora St, Liverpool，生日是1969年2月3日。）

POLICE: Right, and what is your occupation?

SUSPECT: Uh, I’m a brickie’s labourer, I work in the construction industry, helping out wherever is needed.（嗯，我是砌砖工，做建筑的，哪有活就到哪干。）

Police: Ah, okay, Ronny. Tell me about your work at the moment then. How long have you been working there? What exactly do you do? Who do you report to?

SUSPECT: Uh, well, a friend of mine used to work there and he knew I was looking for a job so he told his foreman and he said ok, ask him to come, we need a labourer, so I did and there I am now, uh, I think I’ve been working there for about 3 years? Uh, I don’t know the full name of the foreman, we call him Jim. （嗯，是这样的，有个朋友以前在那工作，他知道我在找工作，就跟工头说了，工头说行叫他来，我们需要工人，于是我就去了，现在还在做，呃，应该在那做了有三年了吧？嗯，我不知道工头全名叫什么，我们都叫他Jim。）

POLICE: Ok, that’s great, thanks. Isn’t it good that your friend recommended you and you got a job? Do you enjoy working there?

SUSPECT: Yeah, it’s a job… （对啊，就是个工作嘛……）

POLICE: Ok, good. And, and could you please tell us your nationality?

SUSPECT: I’m Chinese, I was born in mainland China. (我是中国籍，在中国大陆出生。)

POLICE: Right, and do you travel back to often?

SUSPECT: Uhm, well, yes, quite often, uh, but I really don’t know what you mean by ‘often’, I mean, I go maybe once a year to see my family, my mum in particular. （嗯，这个，对啊，还满常的，嗯，我不太清楚你说的经常具体是什么意思，一般我是每年回去一次看看家人，主要是看我妈。）

POLICE: That’s good that you go and see your mum often. I should go and visit my mum more myself!

And do you travel to other countries apart from China? You travel to other Asian countries as well quite often, don’t you? (42)?

SUSPECT: Uh, well, yes. Why? Is that illegal? （嗯，这个，对啊。干嘛？这也犯法？）

POLICE: No, that’s not illegal. We just need to know what other countries you travel to and the reasons why.

SUSPECT: Well, why? don’t I have the bloody right to visit other countries! ? （可是为什么啊？难道我他妈没权利去其他国家？）

Police: Mr Chen, I ask the question. What other Asian countries have you travelled to in the past twelve months?

Suspect: Well, yes, in the past 12 months I’ve travelled to other countries, I’ve been to Thailand, Laos, Vietnam, Taiwan, Indonesia, lots of places, I love travelling. Have you ever been to Asia yourself?（好吧，对啊，这一年我去过其他国家，我去过泰国、老挝、越南、台湾、印尼，很多地方。我喜欢旅行。你去过亚洲吗？）

Police: No, actually, I’d love to travel and visit those countries! Now, I’m curious. How, how did you get the money to travel so much on a brickies labourer’s income?

SUSPECT: Well, uh, I’ve been saving the money. I live on my own and don’t spend much money. Actually, that’s all I spend my money on, what else is there for a single man to do? （这个嘛，嗯，我一直在存钱。我自己一个人住，花销不大。其实我的钱都花在这上面了，我一个单身汉还能干嘛？）

***SPLIT HERE – (approx. 1000 words) – Police stops the interview***

POLICE: You see, Ronny, your story is a bit unlikely, because you came back to Australia after visiting each of these countries, and you only stayed at each of them for less than a week. And then you go back again after a week or so in Australia.

SUSPECT: Yes, that’s because I had to come back to Australia to work. I couldn’t take more than a week off at a time, and I need to earn more money to cover all the expenses. （对啊，那是因为我得回澳洲继续工作。每次请假不能超过一个礼拜，而且我还得回来继续挣钱付旅行费用呢。）

*(To the interpreter)*: I don’t like the way he’s asking these questions, he’s trying to insinuate that I’ve done something wrong, and that’s not true. Don't tell him this, but I need to talk to you because you speak my language and you can help me. （他这样问我好难受，他在暗示我做了坏事，我才没有。你别告诉他，我只是和你聊一聊，我们都说中文嘛，你帮帮我。）

POLICE: *(The police will respond differently depending on what the interpreter did with the previous segment)*

*Option 1 –* if the interpreter interpreted everything faithfully:

Mr Chen, I warn you that you can’t engage the interpreter in conversation. The interpreter is impartial and is here to interpret everything that is said by you and by me.

*Option 2 –* if the interpreter doesn’t interpret everything and it is obvious:

Sorry interpreter, can you please make sure you interpret everything that is said?

POLICE: Now, Ronny, do you know a Mr Ahmad Ayoub who resides in Australia?

POLICESuspect: Yes, of course, he works with me on the building site, he’s a labourer like me. （知道，当然知道，他跟我一起在建筑工地工作，跟我一样也是工人。）

POLICE: And you’re good mates, aren’t you?

SUSPECT: yeah, pretty much. Although we don’t understand each other very well, because neither of us speaks English very well. He speaks Arabic and I speak Chinese, we just work on the same construction sites, but I wouldn’t say we have much in common with each other. Sometimes we have lunch together. （对啊，没错。不过我们两个人沟通起来也不方便，因为我们英文都不好。他说阿拉伯语我说中文，只是在同一个工地工作而已，也太多共同点。有时候一起吃午饭。）

POLICE: And did you ever talk about politics or religion? Did he ever tell you what his plans were for the future?

SUSPECT: Ah, no, not really. He used to say he didn’t like Australia much and that he wanted to go back home to help his people, so he was working hard to save money to help the poor people in his country, but I don’t really know what he meant by that. I think he was Muslim, but I don't really know. I didn’t care what religion he was. I don't want any trouble, you know? （嗯没有，没怎么说。他以前说过不太喜欢澳洲，想回国去帮助他的同胞，所以他才努力工作存钱帮助他祖国的穷人，但我也不知道他到底是什么意思。我觉得他是穆斯林吧，但也不确定。我才不管他是信什么宗教的。我可不想找麻烦，你知道吧？）

Police: Ronny, we don’t want you to get into any trouble either. Now, did you know that he went to Syria to fight in the jihadist insurgency? (26)?

SUSPECT: Uh, no, I can’t believe that, are you sure? I didn’t think he was that type of person. Are you saying he’s a terrorist? Gee that’s scary! You need to believe me, I didn't know anything! Now I understand why he hasn’t turned up to work for a while. （不知道啊，难以置信，你说真的？我可没看出来他是那种人。你是说他是恐怖分子？天啊太吓人了！你得相信我，我什么都不知道！现在我才明白为什么他好长时间没来上班了。）

Police: But, Ronny, I think you knew him better than what you want to admit. Didn’t he keep in touch with you? Didn’t he send you emails from there?

SUSPECT: Uh, well, I didn’t know where he was sending them from, and everyone gets unwanted emails from all over the place. I often get emails from Nigeria and Russia asking me for money, I’m sure you do too, that doesn’t mean I’m connected to them. （嗯，这个，我根本不知道他从哪给我发的邮件，每个人都常收到乱七八糟的邮件啊，我还经常收到尼日利亚和俄罗斯发来的邮件跟我要钱呢，你肯定也收过嘛，这根本不能说明我跟他们有关系。）

Police: Ok, Ronny, yes, we all do, don’t we? Now, Now, Ronny, I believe you are a Facebook friend of Ahmad? Aren’t you?

SUSPECT: Uh, well, yes, I think so, I don't know really. I can’t keep track of all the Facebook friends, most of them I don’t know, they’re friends of friends. I was thinking of getting rid of this bloody Facebook account. It’s nothing but a nuisance, and now this confirms it! （这个嘛，是啊，应该是，我也不太确定。我又不记得所有的Facebook朋友都有谁，反正大部分都不认识，都是朋友的朋友。之前我还想删了这该死的Facebook，除了麻烦没啥用，现在好了吧，果然给我惹麻烦了！）

POLICE: And, have you seen his latest posts on his wall where he’s posted photos of himself in jihadist uniform fighting in Syria?

SUSPECT: Uh, no, frankly I haven’t been on Facebook for a while（啊没哪，我好久没上Facebook了。）

POLICE: Ok. Now, do you know a Mr Huang who resides in China?

SUSPECT: Yes, I know him （对，我认识他。）

POLICE: And, can you tell me about how you know him? (10)

SUSPECT: Well, uh, he was introduced to me by another friend who travelled with me to China last time. Why? Is he in Syria as well? （是这样的，嗯，他是上次跟我一起回国的一个朋友介绍的。怎么了？他也去叙利亚了？）

POLICE: We have reason to believe that he has contacts in Syria through you. Have you introduced Huang to Ahmad?

SUSPECT: No….no…Why would I? They don’t even speak the same language or belong to the same religion（没有，没有，干嘛介绍？他们说的话都不一样，又不是一个地方来的。）

POLICE: That’s why you are useful to them, Ronny, because you can sort of translate for them.

SUSPECT: No way! How can I translate when I can hardly speak English! （不可能！我连英文都不怎么会说，怎么可能翻译？）

POLICE: You see Ronny, we have reason to believe that you may be part of a money laundering cell in Australia

SUSPECT: No way! I don’t even know what that means! （不可能！我都不知道你说的是什么意思！）

*– To the interpreter –* Can you please tell him I’m innocent? I can’t stand this for much longer! I want to go home now. They can’t keep me here like this. （请你跟他说我是清白的！我受不了啦！我现在就想回家。他们不能一直让我待在这。）

POLICE: Now, Ronny, please calm down. I can tell you that the Chinese drug police has raided Huang’s house and confiscated $10,000 worth of mythelamphetamine. Did you know about that?

SUSPECT: No… how would I know that? I told you I don’t know that bloke well（不知道……我怎么会知道？我跟你说了我跟那个人不熟。）

Police: Then how do you explain that among his bank transactions there was one made to your bank account for $100,000? And futhermore, that you then made a transfer to Ahmad’s account for $90,000?

SUSPECT: Uh, look, I’m not feeling well now and I think I can’t answer any more questions, I think I need to call a solicitor, but I don’t know who to call （我说，现在我感觉很不舒服，不能再回答问题了，我应该打电话给律师，但我又不知道打给谁。）

POLICE: Ok Ronny, we’ll suspend the interview now to let you find a solicitor. Here’s a list of solicitors available to you for the purpose of providing advice. Please choose one and we will make arrangements to put you in touch with the solicitor of your choosing.

SUSPECT: Look, in that case, let’s just finish the interview, because I want to finish now. I don’t know who to call. （那这样的话，就继续问完吧，我现在就想停，反正我也不知道要打给谁。）

POLICE: Ok, then. Mr Chen, I am now formally charging you with being knowingly involved in money laundering with the purpose of aiding and abetting terrorist activity. You will be summoned to go to court to answer the charge where you can plead guilty or not guilty.

SUSPECT: No, no, no, I can’t believe this! What have I got myself into?! （别别别，怎么可能！我这都弄了什么呀？！）

POLICE: Now, this concludes the interview. Have you given your answers of your own free will and choice?

SUSPECT: Yes, yes.（是，是。）

POLICE: Has anyone made any threat, promise or inducement to give your answers?

SUSPECT: No, no, please let me go now. （没有没有，请让我走吧。）

POLICE: Ok, Ronny, that concludes the interview. Thank you Madam/Mr Interpreter for your excellent work.

SUSPECT: Ok. Thank you. （好吧，谢谢。）

(End of script)
