# Supplementary material for: An Eye-Movement Analysis of Overt Visual Attention During Consecutive and Simultaneous Interpreting Modes in a Remotely Interpreted Investigative Interview
Source: Front Psychol. 2022 Mar 25;13:764460. doi: 10.3389/fpsyg.2022.764460 (PMC8992695; doi:10.3389/fpsyg.2022.764460)
Supplement: Supplementary file 4 [file Data_Sheet_4.doc]

Police interview

POLICE: (To interpreter) Hello Mr/Madam Interpreter. Thank you for coming. We’ll be interviewing this man who we understand speaks only Spanish and can’t speak English, so we need you to interpret for us. Can you please introduce yourself and explain your role to him? (46)

*Interpreter: The interpreter is expected to explain her/his role here to both parties. The next turn will need to be adapted according to what the interpreter says.*

SUSPECT: Bueno, qué bien, gracias por venir, estaba preocupado de que no me iba a poder comunicar con la policía, y sé que eso puede ser bastante peligroso. (27)

POLICE: Ok, thank you. My name is Detective Inspector Costa and I’m attached to the Joint Counter Terrorism Team. We also have a Spanish interpreter with us, who will interpret everything from and into English. Are you able to understand the interpreter? (42)

SUSPECT: Sí, claro, la entiendo perfectamente. (5)

POLICE: First of all, I have to tell you that we need to ask you certain questions, so I must caution you that you don’t have to say or do anything, but everything that you do say or do will be recorded and may be used in evidence. Do you understand? (50)

SUSPECT: ¿Cómo? ¿Qué me está diciendo? ¿Qué? ¿ya me están mandando a la corte?, pero ¿por qué? ¿Qué he hecho yo? Yo quiero muchas respuestas, porque yo no he hecho nada malo, así que no entiendo por qué estoy en esta situación tan incómoda. Estoy tan cansado después del viaje tan largo que me quiero ir a casa ahora mismo. (59)

POLICE: Before we continue, you say that you are very tired. Are you ok to continue with the interview now? Or do you need some time to rest? (28)

SUSPECT: No, no, me quiero ir lo antes possible, por favor continue así me puedo marchar. (15)

POLICE: Ok, good. But first it is very important for me to know that you understand the official caution I have given you. Can you please explain it back to me in your own words? (34)

SUSPECT: Bueno, sí, que no tengo que decir nada que no quiera y que lo que diga se podrá usar en el tribunal ¿no? (23)

POLICE: Yes, that’s right. Now I will tell you what your rights are: You have the right to legal representation. Would you like to contact a lawyer? (26)

SUSPECT: Bueno, eh, no, no tengo abogado. (6)

POLICE: Ok. You also have the right to speak with a consular official or a support person, a relative or friend. Would you like to contact anyone before we start? (29)

SUSPECT: No, no, está bien, ¡siga no más, por favor! ¡Esto me está poniendo cada vez más nervioso! (16)

POLICE: Ok, no need to get nervous. If at any time during the interview you decide that you want a lawyer, friend, relative or consular official, please let me know and we will suspend the interview and help you contact one. Ok? (41)

SUSPECT: Bueno, muy amable. (3)

POLICE: Also, there is a video camera over there recording everything. At the conclusion of the interview you will be provided with a copy of the recording. (27)

SUSPECT: Ah, bueno, qué bien, por si lo necesito. (8)

POLICE: If at any time you don’t understand a question that’s been asked, please let us know, ok? Also, if you ever need a break, water or food, let me know. And are you well enough to continue the interview? (62)

SUSPECT: Ah, bueno, yo le digo si no entiendo algo, y no se preocupe que estoy bien, sigamos no más. (19)

POLICE: Ok, thank you. Now I can tell you that we’re investigating your involvement in a transnational criminal syndicate, which is believed to be financing a terrorist organisation. (51)

SUSPECT: ¿Qué? ¡Eso es ridículo! Yo no tengo nada que ver con nada que sea ilegal. (14)

POLICE: Now, can I ask you to please state your full name, address and date of birth? (16)

SUSPECT: Bueno, me llamo Ronaldo González pero todo el mundo me llama Ronny, y ahora vivo en 3/15 Flora St, Liverpool y nací el 3/2/1969. (24)

POLICE: Right, and what is your occupation? (6)

SUSPECT: Eh, soy peón de albañil, trabajo en la construcción, ayudo donde me necesiten. (13)

POLICE: Ah, okay, Ronny. Tell me about your work at the moment then. How long have you been working there? What exactly do you do? Who do you report to? (29)

SUSPECT: Eh, bueno, un amigo mio que trabajaba ahí y sabía que yo estaba buscando trabajo le dijo a su capataz y le dijo que sí, que me dijera que vaya, que necesitaban un peón, así que fui y aquí me tienen, eh… creo que ya llevo 3 años trabajando ahí. Eh, el nombre del capataz es Jim, pero la verdad que no sé el apellido. (65)

POLICE: Ok, that’s great, thanks. Isn’t it good that your friend recommended you and you got a job? Do you enjoy working there? (22)

SUSPECT: Bueno, sí, es un trabajo…(5)

POLICE: Ok, good. And could you please tell us your nationality? (10)

SUSPECT: Soy colombiano, nací en Colombia. (5)

POLICE: Right, and do you travel back to South America often? (10)

SUSPECT: Eh, bueno, sí, bastante, eh, pero en realidad no sé lo que quiere decir cuando dice “a menudo”, o sea, voy una vez al año a ver a mi familia, a mi madre en particular que está bastante viejita y no muy bien de salud. (45)

POLICE: That’s good that you go and see your mum often. I should go and visit my mum more myself! And do you travel to other countries apart from Colombia? You travel to other Latin American countries as well quite often, don’t you? (42)

SUSPECT: Eh, bueno, sí, ¿por qué? ¿Es ilegal eso? (8)

POLICE: No, that’s not illegal. We just need to know what other countries you travel to and the reasons why. (19)

SUSPECT: Bueno, ¿por qué? ¿No tengo el ‘puto’ derecho de visitar otros países? (11)

POLICE: Mr Gonzalez, I ask the questions. What other South American countries have you travelled to in the past twelve months? (20)

SUSPECT: Bueno, sí, en los últimos 12 meses he viajado a otros países, he visitado Venezuela, México, Chile, Argentina, Perú, muchos países, me encanta viajar, especialmente Sud América. ¿Usted ha estado en Sud América alguna vez? (35)

POLICE: No actually, I’d love to travel and visit all those countries!

Now, I’m curious. How did you get the money to travel so much on a brickies labourer’s income? (29)

SUSPECT: Bueno, eh, he estado ahorrando dinero. Yo vivo solo y no gasto mucho dinero, de hecho en lo único que gasto es en viajar, ¿en qué otra cosa voy a gastar el dinero si vivo solo? (36)

581 Police words

420 suspect words

Total 1001

***SPLIT HERE – (approx. 1000 words) – Police 2 stops the interview***

POLICE: You see, Ronny, your story doesn’t quite add up, because you came back to Australia after visiting each of these countries, and you only stayed at each of them for less than a week. And then you go back again after a week or so in Australia. (48)

SUSPECT:Sí, es porque tengo que volver a Australia a trabajar. No me puedo tomar más de una semana de vacaciones y además necesito ahorra más dinero para cubrir todos los costos (39)

*(To the interpreter)*: Mire, la verdad es que no me gusta cómo me está preguntando estas preguntas, está tratando de insinuar que yo he hecho algo malo, y eso no es verdad. No le diga esto, pero necesito hablar con ud porque usted habla mi idioma y me puede ayudar. (47)

POLICE: *(respond differently depending on what the interpreter did with the previous segment)*

*Option 1 –* if the interpreter interpreted everything faithfully:

Mr Gonzalez, I warn you that you can’t engage the interpreter in conversation. The interpreter is impartial and is here to interpret everything that is said by you and by me. (31)

*Option 2 –* if the interpreter doesn’t interpret everything and it is obvious:

Sorry interpreter, can you please make sure you interpret everything that is said? (13)

POLICE: Now, Ronny, do you know a Mr Ahmad Ayoub who resides in Australia? (13)

SUSPECT: Sí, claro, él trabaja conmigo en la construcción, es peón de albañil como yo. (14)

POLICE: And you’re good mates, aren’t you? (6)

SUSPECT: Sí, más o menos. aunque no nos entendemos muy bien porque ninguno habla inglés, él habla árabe y yo español, solo que trabajamos juntos en las obras, pero la verdad es que no tenemos mucho en común, a veces almorzábamos juntos. (41)

POLICE: And did you ever talk about politics or religion? Did he ever tell you what his plans were for the future? (21)

SUSPECT: Bueno, eh, él solía decir que no le gustaba Australia y que se quería volver a su país, a su gente, entonces trabajaba muchas horas para ahorrar dinero para ayudar a los pobres en su país. Creo que es musulmán, pero la verdad que no lo sé tampoco. A mí no me importa de qué religión es. Yo nunca quise ningún problema, ¿sabe? (61)

POLICE: Ronny, we don’t want you to get into any trouble either. Now, did you know that he went to Syria to fight in the jihadist insurgency? (26)

SUSPECT: ¡No! No lo puedo creer, ¿está seguro? Yo no pensaba que fuera esa clase de gente. ¿Me está diciendo que es un terrorista? Bueno, ¡qué miedo! Usted me tiene que creer que yo no sabía nada. ¡Ahora entiendo por qué hace mucho que no va a trabajar! (47)

POLICE: But, Ronny, I think you knew him better than what you want to admit. Didn’t he keep in touch with you? Didn’t he send you emails from there? (28)

SUSPECT: Eh, bueno, yo no sabía de dónde los enviaba, y todos recibimos emails que no queremos de todo el mundo. Yo a veces recibo emails de Nigeria y de Rusia que me piden dinero, y seguro que a ud también le habrá pasado lo mismo, eso no significa que yo esté conectado con esa gente. ¿No? (55)

POLICE: Ok, Ronny, yes, we all do, don’t we? Now, Ronny, I believe you are a Facebook friend of Ahmad? Aren’t you? (21)

SUSPECT: Eh, bueno, sí, creo, pero en realidad no sé. No puedo saber cuántos amigos tengo en Facebook, la mayoría son amigos de amigos. Para serle franco, estaba pensando borrar la maldita cuenta de Facebook porque no sirve para nada más que dolores de cabeza, y ¡esto lo confirma! (45)

POLICE: And, have you seen his latest posts where he’s uploaded photos of himself in jihadist uniform fighting in Syria? (22)

SUSPECT: Eh, no, la verdad que hace mucho que no entro a Facebook. (12)

POLICE: Ok. Now, do you know a Mr Pedro López who resides in Colombia? (13)

SUSPECT: Sí, lo conozco, sí. (4)

POLICE: And, can you tell me about how you know him? (10)

SUSPECT: Bueno, eh, a él me lo presentó otro amigo que viajó conmigo a Colombia la última vez que fui. ¿Por qué? ¿él también está en Siria? (26)

POLICE: We have reason to believe that he has contacts in Syria through you. Have you introduced Lopez to Ahmad? (19)

SUSPECT: No…No…¿Para qué los iba presentar? Ni hablan el mismo idioma ni son de la misma religión. (16)

POLICE: That’s why you are useful to them, Ronny, because you can sort of translate for them. (16)

SUSPECT: ¿Yo? ¿Traducir? ¿Cómo puede ser eso si casi no hablo inglés? (11)

POLICE: You see Ronny, we have reason to believe that you may be part of a money laundering cell in Australia. (20)

SUSPECT: ¡No, para nada! ¡Ni siquiera sé lo que eso significa! (10)

*– To the interpreter –* ¿Le puede decir por favor que soy inocente? ¡Ya no puedo más con esto! Me quiero ir a casa ¡No me pueden detener aquí de esta manera! (27)

POLICE: Now, Ronny, please calm down. I can tell you that the Colombina drug police raided Pedro’s house and confiscated $10,000 worth of mythelamphetamine. Did you know about that? (28)

SUSPECT: No…¿por qué iba a hacer eso? Ya le dije que no conozco bien a ese tipo. (16)

POLICE: Then how do you explain that among his bank transactions there was one made to your account for $100,000? And futhermore, that you then made a transfer to Ahmad’s account for $90,000? (33)

SUSPECT: Eh, mire, no me estoy sintiendo muy bien y creo que ya no puedo seguir contestando más preguntas, creo que necesito llamar a un abogado, pero no sé a quién llamar (31)

POLICE: Ok Ronny, we’ll suspend the interview now to let you find a solicitor. Here’s a list of solicitors available to you for the purpose of providing advice. Please choose one and we will make arrangements to put you in touch with the solicitor of your choosing. (46)

SUSPECT: Bueno, entonces en ese caso, no. Terminemos la entrevista, porque quiero terminar ahora. No sé a quién llamar. (18)

POLICE: Ok, then. Mr Gonzalez, I am now formally charging you with being knowingly involved in money laundering with the purpose of aiding and abetting terrorist activity. You will be summoned to go to court to answer the charge where you can plead guilty or not guilty. (46)

SUSPECT: ¡No, no, no! ¡No lo puedo creer! ¡En qué me he metido! (12)

POLICE: Now, this concludes the interview. Have you given your answers of your own free will and choice? (19)

SUSPECT: Sí, sí. (2)

POLICE: Has anyone made any threat, promise or inducement for you to give your answers? (12)

SUSPECT: No, no, por favor, déjeme ir ahora. (7)

POLICE: Ok, Ronny, that concludes the interview. Thank you Madam/Mr Interpreter for your excellent work. (14)

SUSPECT: Bueno, muchas gracias. (3)

(End of script)

Police words: 477

Suspect words: 542

Total words: 1019

Total number of words: 2020
