# Supplementary material for: An Eye-Movement Analysis of Overt Visual Attention During Consecutive and Simultaneous Interpreting Modes in a Remotely Interpreted Investigative Interview
Source: Front Psychol. 2022 Mar 25;13:764460. doi: 10.3389/fpsyg.2022.764460 (PMC8992695; doi:10.3389/fpsyg.2022.764460)
Supplement: Supplementary file 5 [file Data_Sheet_5.docx]

Interpreting Performance Assessment Criteria

| **Element of competence / coded variable** | **Criterion descriptors** | **Mark** | **Weight** |
| --- | --- | --- | --- |
| Accuracy of propositional content | The interpreter maintains the content of the utterance, 'what' the speaker said. | 10 | 30% |
| Accuracy of style | The interpreter maintains stylistic features, the 'how' of the utterance. This includes pragmatic force (tone, intonation, stress, hesitations, fillers, hedges, repetitions, etc.) and maintenance of register (formal/informal, technical/colloquial). | 10 | 15% |
| Maintenance of verbal rapport markers | The interpreter maintains the rapport features of the original. These include use of first name, acknowledgement markers such as ‘OK’ at the start of a response, politeness markers such as ‘please’ and ‘thank you’, expressions of solidarity and comfort. | 10 | 15% |
| Use of correct interpreting protocols | The use of the direct approach (1^st^ & 2^nd^ grammatical persons), interpreting everything that is said regardless of what it is, seeking repetitions when needed in the right way, transparency (keeping everyone informed if a repetition or clarification is required). | 10 | 10% |
| Legal discourse and terminology | Maintaining institutional phrases and grammatical structures, correct use of strategic question types, legal formulas and correct legal terminology. | 10 | 10% |
| Management and coordination skills | This includes setting the contract by establishing the interpreter's role and modus operandi, knowing when to interpret and how to manage the interaction. | 10 | 10% |
| Language competence | Grammatical correctness, correct pronunciation, fluency in both languages | 10 | 10% |
| Total mark |  | 70 | 100% |
